# Supplementary material for: Reversible modulation of interlayer stacking in 2D copper-organic frameworks for tailoring porosity and photocatalytic activity
Source: Nat Commun. 2024 Jan 3;15:194. doi: 10.1038/s41467-023-44552-w (PMC10764794; doi:10.1038/s41467-023-44552-w)
Supplement: Supplementary file 1 — Supplementary Information [file 41467_2023_44552_MOESM1_ESM.pdf]

Supplementary Information

Reversible Modulation of Interlayer Stacking in 2D Copper-Organic Frameworks for Tailoring Porosity and Photocatalytic Activity

Pei-Ye You,[a] Kai-Ming Mo,[a] Yu-Mei Wang,[a] Qiang Gao,[b] Xiao-Chun Lin,[a] Jia-Tong Lin,[a] Mo Xie,[a] Rong-Jia Wei,[a] Guo-Hong Ning[a,]* and Dan Li[a,]*

[a] College of Chemistry and Materials Science, and Guangdong Provincial Key Laboratory of Functional Supramolecular Coordination Materials and Applications, Jinan University, Guangzhou, 510632, People's Republic of China

[b] CAS Key Lab of Low-Carbon Conversion Science and Engineering, Shanghai Advanced Research Institute, Chinese Academy of Sciences, Shanghai 201210, People's Republic of China

*Corresponding authors: guohongning@jnu.edu.cn; danli@jnu.edu.cn;

Contents

1. General procedure 3
2. Synthesis of JNMs 4
2.1 Synthesis of Cu-CTU 4
2.2 Synthesis of **JNM-7-AA, JNM-8-AA and JNM-9-ABC**. 4
3. Characterization of JNM-7-AA, JNM-8-AA and JNM-9-ABC. 7
3.1 Structural simulation 7
3.2 Energy Dispersive X-ray Spectroscopy (EDS) 19
3.3 Gas adsorption isotherms and pore size distribution of JNM-9-ABC. 20
4. Reversible Structure transformation 21
4.1 Experimental procedures 21
4.2 Structural simulation for JNM-7-ABC and JNM-8-ABC 22
4.3 Reversible structure transformation monitoring by PXRD. 26
4.4 Fourier-transform infrared (FT-IR) spectra 28
4.5 Solid-state ^{13}C CP/MAS NMR spectra 28
4.6 Scanning electron microscopy (SEM). 28
4.7 X-ray photoelectron spectroscopy (XPS). 29
4.8 Thermogravimetric analysis (TGA) 29
4.9 Various-Temperature PXRD. 30
4.10 Stability in various solvents. 30
4.11 Gas adsorption isotherms and pore size distribution of JNM-8. 31
4.12 DFT calculations[2, 3] 31
4.13 Reversible encapsulation and release of lipase 32
5. Optical and electronic properties 35
6. Photocatalytic Studies 37
6.1 Experiments of CDC reactions. 37
6.2 Characterization for the product of JNM-8-AA catalyzed CDC reaction. 37
6.3 Gram-scale CDC reactions. 38
6.4 Comparison of yield and TOF for JNM-8-AA with existing catalysts. 39
6.5 Catalytic recyclability for JNM-8-AA. 40
6.7 Scope of JNM-8-AA Catalyst for CDC reactions. 42
^{1}H NMR and ^{13}C NMR spectra of all compounds 45
Supplementary references 53

1. General procedure

Starting materials, reagents, and solvents were purchased from commercial sources and used without further purification. Powder X-ray diffraction (PXRD) data was collected at 40 kV, 30 mA using microcrystalline samples on a Rigaku Ultima IV diffractometer using Cu-Kα radiation (λ = 1.5418 Å). The measurement parameters include a scan speed of 0.5°/min, a step size of 0.02°, and a scan range of 2θ from 1.5° to 30°. For Temperature-dependent PXRD, the measurement parameters include a scan speed of 2 °C/min, a step size of 0.02°, and a scan range of 2θ from 1.5° to 30°. Thermogravimetric analysis was performed on a Mettler-Toledo (TGA/DSC1) thermal analyzer. Measurement was made on approximately 5 mg of dried samples under an N_2 flow with a heating rate of 10 °C/min. The scanning electron microscopy (SEM) images and Energy Dispersive X-ray Spectroscopy (EDS) were obtained on a JEOLJSM7600F microscope, operating at an acceleration voltage of 5 kV and magnification is 2000. Transmission electron microscopy (TEM) analysis was performed on an FEI Titan 80-300 S/TEM (Scanning /Transmission Electron Microscope) operated at 200 kV. Fourier transform infrared (FT-IR) spectrum was measured using a Nicolet Avatar 360 FT-IR spectrophotometer. X-ray photoelectron (XPS) spectroscopy spectra were performed by a Thermo ESCALAB 250XI system. GC-MS analysis was carried out on an Agilent 7890B GC analyzer. The ^{1}H and ^{13}C NMR spectra were recorded on Bruker Biospin Avance (400 MHz) equipment using tetramethylsilane (TMS) as an internal standard. Solid-state NMR experiments were performed on a Bruker WB Avance II 400 MHz NMR spectrometer. The ^{13}C CP/MAS NMR spectra were recorded with a 4-mm double-resonance MAS probe and with a sample spinning rate of 10.0 kHz; a contact time of 2 ms (ramp 100) and a pulse delay of 3 s was applied. Gas sorption analyses were conducted using an ASAP 2020 PLUS Analyzer (Micromeritics) with extra-high pure gases. The samples were outgassed at 100 ºC for 8 h before the measurements. Surface areas were calculated from the adsorption data using Brunauer-Emmett-Teller (BET) methods. The pore size distribution curves were obtained from the adsorption branches using the density functional theory (DFT) method.

2. Synthesis of JNMs

2.1 Synthesis of Cu-CTU

Cu-CTU was synthesized according to the previously reported method.[1] A mixture of the ligand 1H-pyrazole-4-carbaldehyde (HL) (24.0 mg, 0.25 mmol), Cu_2O (14.3 mg, 0.1 mmol), 4 mL ethanol, and 0.1 mL pyridine was sealed in an 10 mL Pyrex tube, heated in an oven at 120 °C for 72 h. The light-yellow needle crystals of Cu-CTU formed were filtered and collected under a microscope manually. The yield of Cu-CTU: 23.7 mg (75.8%, based on Cu_2O). Chemical formula: $C_{12}H_9Cu_3N_6O_3$.

2.2 Synthesis of JNM-7-AA, JNM-8-AA and JNM-9-ABC.

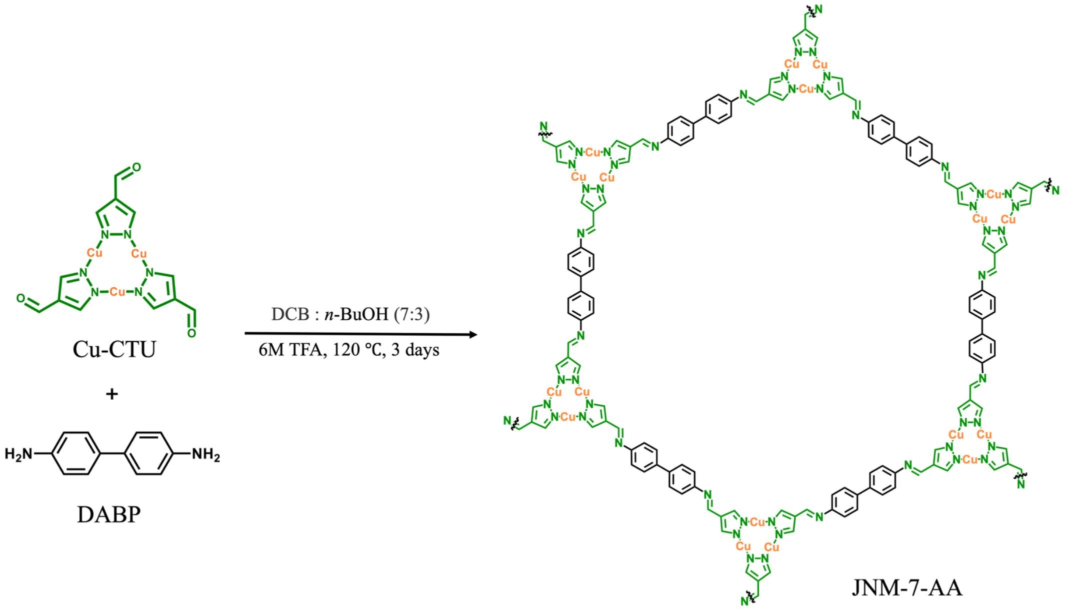

JNM-7-AA: a 10 mL Schlenk tube was charged with Cu-CTU (23.7 mg, 0.05 mmol), *p*-4,4'-diaminobiphenyl (DABP) (13.8 mg, 0.075 mmol), 0.7 mL of 1,2-dichlorobenzene, 0.3 mL of 1-butanol, and 0.1 mL of 6 M trifluoroacetic acid. Schlenk tube containing was flash-frozen at 77 K in a liquid nitrogen bath and degassed with three freeze-pump-thaw cycles. Upon warming to room temperature, tube was heated at 120 °C for 72 h. The brown solid from each tube was isolated by filtration, washed, and the solvent exchanged with methanol, ethanol and dichloromethane. The resultant solids were dried under vacuum at 100 °C for 8 h to give JNM-7-AA as brown powders. Yield: 21.5 mg (70.6% based on Cu). IR (KBr): ν = 1668 (m), 1625 (s), 1592 (m), 1541 (m), 1488 (s), 1403 (w), 1376 (w), 1315 (w), 1201 (m), 1139 (w), 1062 cm^{-1} (w).

Elemental analysis for $C_{60}H_{42}N_{18}Cu_6{\cdot}2C_6H_4Cl_2{\cdot}11H_2O$, calcd (%): C 45.79, H 3.84, N 13.35; Found (%): C 42.04, H 2.80, N 13.82. The theoretical pore size of JNM-7-AA is ~3.5 nm.

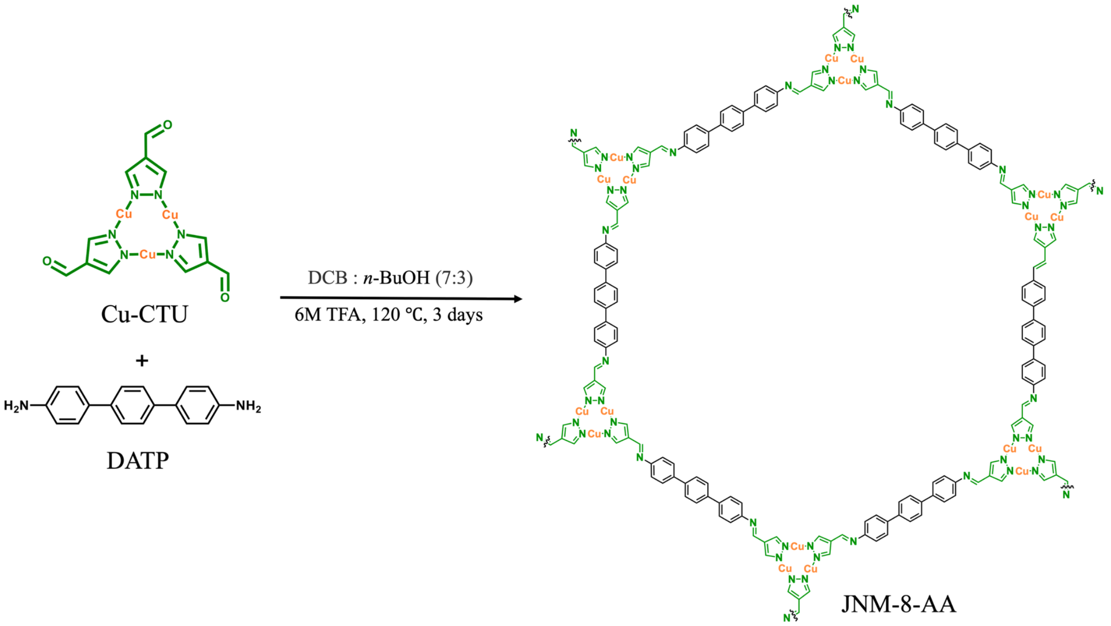

JNM-8-AA: a 10 mL Schlenk tube was charged with Cu-CTU (23.7 mg, 0.05 mmol), *p*-4,4'-diamino-*p*-terphenyl (DATP) (19.5 mg, 0.075 mmol), 0.5 mL of 1,2-dichlorobenzene, 0.5 mL of 1-Butanol, and 0.1 mL of 6 M trifluoroacetic acid. Schlenk tube containing was flash-frozen at 77 K in a liquid nitrogen bath and degassed with three freeze-pump-thaw cycles. Upon warming to room temperature, tube was heated at 120 °C for 72 h. The brown solid from each tube was isolated by filtration, washed, and the solvent exchanged with methanol, ethanol and dichloromethane. The resultant solids were dried under vacuum at 100 °C for 8 h to give JNM-8-AA as brown powders. Yield: 35.0 mg (97% based on Cu). IR (KBr): ν = 1666 (m), 1625 (s), 1589 (m), 1545 (m), 1483 (s), 1393 (w), 1375 (w), 1316 (w), 1207 (m), 1171 (m), 1058 (m), 1002 cm^{-1} (m). Elemental analysis for $C_{78}H_{54}N_{18}Cu_6{\cdot}3C_6H_4Cl_2{\cdot}10H_2O$, calcd (%): C 51.34, H 3.86, N 11.23; Found (%): C 48.67, H 2.12, N 11.13. The theoretical pore size of JNM-8-AA is ~4.4 nm.

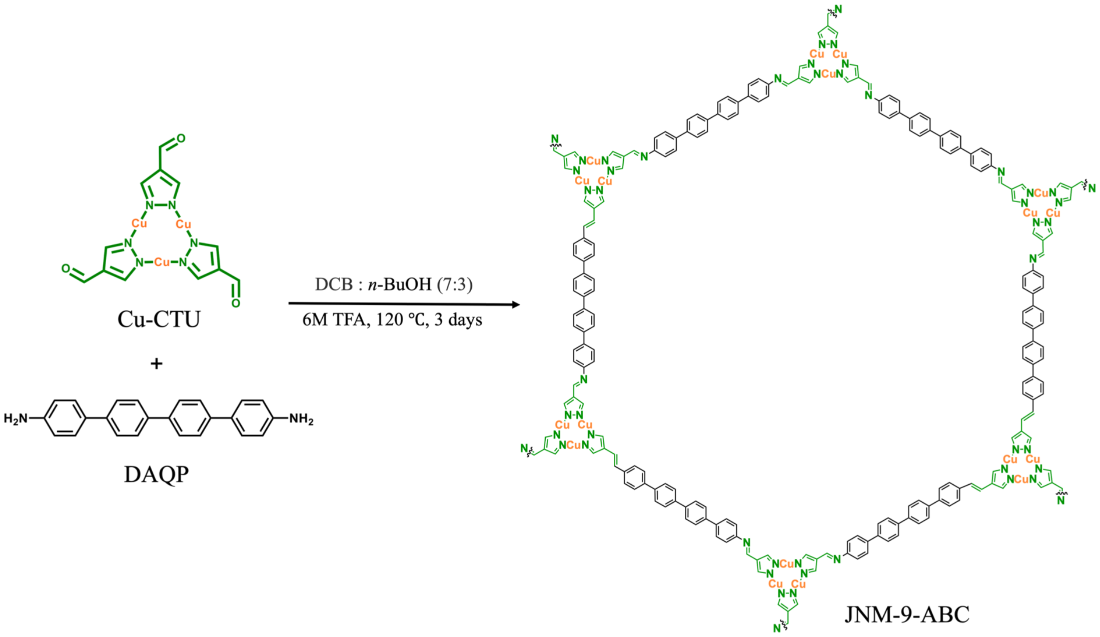

JNM-9-ABC: a 10 mL Schlenk tube was charged with Cu_3L_3 (23.7 mg, 0.05 mmol), 4,4'-diamino-*p*-quaterphenyl (DAQP) (25.2 mg, 0.075 mmol), 0.3 mL of 1,2-dichlorobenzene, 0.7 mL of 1-Butanol, and 0.1 mL of 6 M trifluoroacetic acid. Schlenk tube containing was flash-frozen at 77 K in a liquid nitrogen bath and degassed with three freeze-pump-thaw cycles. Upon warming to room temperature, tube was heated at 120 °C for 72 h. The pale-yellow solid from each tube was isolated by filtration, washed, and the solvent exchanged with methanol, ethanol and dichloromethane. The resultant solids were dried under vacuum at 100 °C for 8 h to give JNM-9-ABC as pale-yellow powders. Yield: 38.5 mg (92% based on Cu). IR (KBr): ν = 1670 (m), 1623 (s), 1589 (m), 1551 (m), 1487 (s), 1404 (w), 1323 (w), 1256 (w), 1193 (m), 1045 cm^{-1} (w). Elemental analysis for $C_{96}H_{66}N_{18}Cu_6 \cdot C_6H_4Cl_2 \cdot 2H_2O$, calcd (%): C 60.17, H 3.66, N 12.38; Found (%): C 60.37, H 3.39, N 11.81. The theoretical pore size of JNM-9-ABC is ~1.9 nm.

3. Characterization of JNM-7-AA, JNM-8-AA and JNM-9-ABC.

3.1 Structural simulation

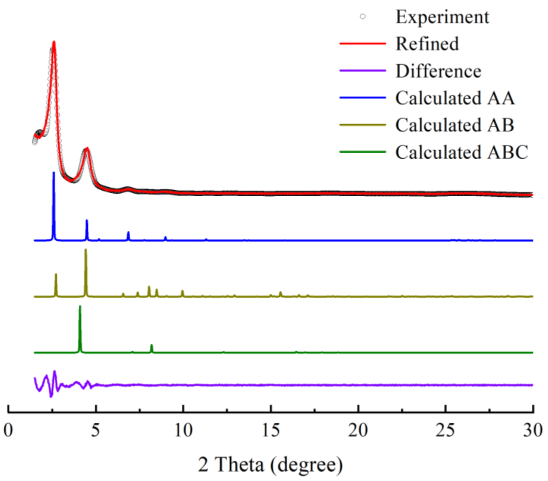

Supplementary Fig 1. PXRD patterns of **JNM-7-AA** with the experimental profiles in red, difference curve in purple, and calculated profiles of AA (blue), AB (yellow), and ABC (green).

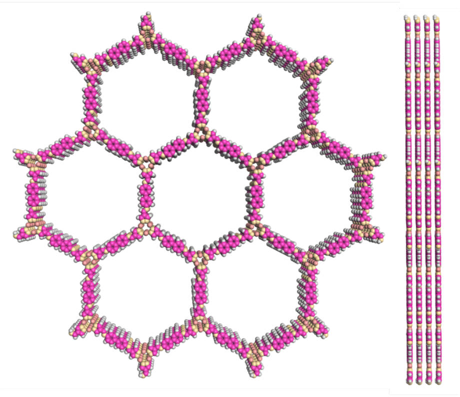

Supplementary Fig 2. Space-filling model of JNM-7-AA in AA stacking mode viewed from (left) *c* axis and (right) *a* axis.

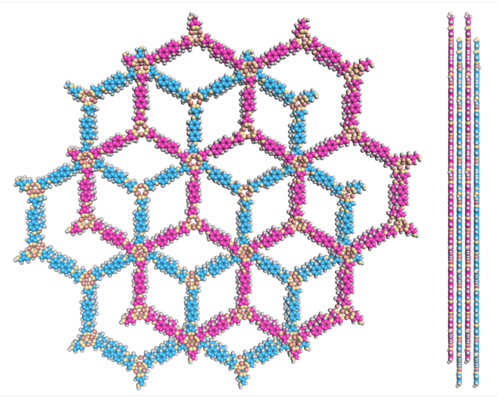

Supplementary Fig 3. Space-filling model of JNM-7-AB in AB stacking mode viewed from (left) *c* axis and (right) *a* axis.

Supplementary Table 1. Fractional atomic coordinates for JNM-7-AA.

JNM-7-AA

Space group symmetry: *P*6/*m*

$a = b = 41.2260$ Å; $c = 3.5549$ Å

$\alpha = \beta = 90.0000°$; $\gamma = 120.0000°$

Atom name	Atom	x	y	z
N1	N	0.37985	0.74181	0.5
C2	C	0.41129	0.61368	0.5
C3	C	0.37241	0.59409	0.5
C4	C	0.43594	0.59767	0.5
C5	C	0.44512	0.54463	0.5
C6	C	0.48438	0.56609	0.5
C7	C	0.50589	0.54854	0.5
C8	C	0.4886	0.50931	0.5
C9	C	0.44932	0.48804	0.5
C10	C	0.42779	0.50557	0.5
N11	N	0.42226	0.56173	0.5
Cu12	Cu	0.31147	0.61267	0.5
N13	N	0.26222	0.60827	0.5
C14	C	0.22852	0.57694	0.5
H15	H	0.35657	0.56216	0.5
H16	H	0.4676	0.61708	0.5
H17	H	0.49884	0.59798	0.5
H18	H	0.53775	0.56623	0.5
H19	H	0.43469	0.45614	0.5
H20	H	0.39591	0.48801	0.5
H21	H	0.22268	0.54704	0.5

Supplementary Table 2. Fractional atomic coordinates for JNM-7-AB.

**JNM-7-AB**				
Space group symmetry: *P*63/*m*				
$a = b = 41.2260$ Å; $c = 6.8000$ Å; $\alpha = \beta = 90.0000°$; $\gamma = 120.0000°$				
**Atom name**	**Atom**	**x**	**y**	**z**
N1	N	0.71318	0.40847	0.25
C2	C	0.74462	0.28035	0.25
C3	C	0.70574	0.26076	0.25
C4	C	0.76927	0.26434	0.25
C5	C	0.77845	0.21129	0.25
C6	C	0.81772	0.23276	0.25
C7	C	0.83922	0.21521	0.25
C8	C	0.82193	0.17598	0.25
C9	C	0.78265	0.15471	0.25
C10	C	0.76112	0.17224	0.25
N11	N	0.75559	0.22839	0.25
Cu12	Cu	0.6448	0.27934	0.25
N13	N	0.59555	0.27493	0.25
C14	C	0.56186	0.2436	0.25
H15	H	0.6899	0.22883	0.25
H16	H	0.80094	0.28374	0.25
H17	H	0.83217	0.26464	0.25
H18	H	0.87109	0.23289	0.25
H19	H	0.76803	0.12281	0.25
H20	H	0.72925	0.15467	0.25
H21	H	0.55601	0.2137	0.25
N22	N	0.95349	-0.07514	0.25
C23	C	0.92204	0.05298	0.25
C24	C	0.96092	0.07258	0.25
C25	C	0.89739	0.06899	0.25
C26	C	0.88822	0.12204	0.25
C27	C	0.84895	0.10058	0.25
C28	C	0.82745	0.11813	0.25
C29	C	0.84473	0.15736	0.25
C30	C	0.88402	0.17863	0.25
C31	C	0.90554	0.16109	0.25
N32	N	0.91108	0.10494	0.25
Cu33	Cu	1.02187	0.05399	0.25
N34	N	1.07112	0.0584	0.25
C35	C	1.10481	0.08973	0.25
H36	H	0.97676	0.10451	0.25
H37	H	0.86573	0.04959	0.25
H38	H	0.83449	0.06869	0.25
H39	H	0.79558	0.10044	0.25
H40	H	0.89864	0.21052	0.25
H41	H	0.93742	0.17866	0.25
H42	H	1.11066	0.11963	0.25

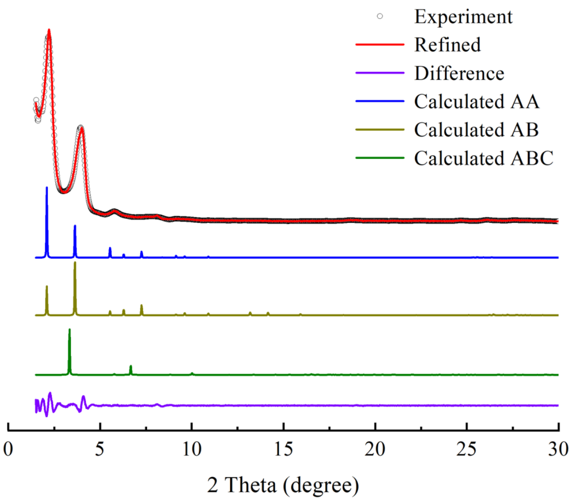

Supplementary Fig 4. PXRD patterns of JNM-8-AA with the experimental profiles in red, difference curve in purple, and calculated profiles of AA (blue), AB (yellow), and ABC (green).

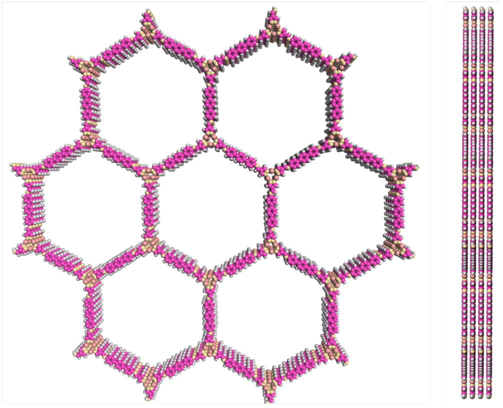

Supplementary Fig 5. Space-filling model of JNM-8-AA in AA stacking mode viewed from (left) *c* axis and (right) *a* axis.

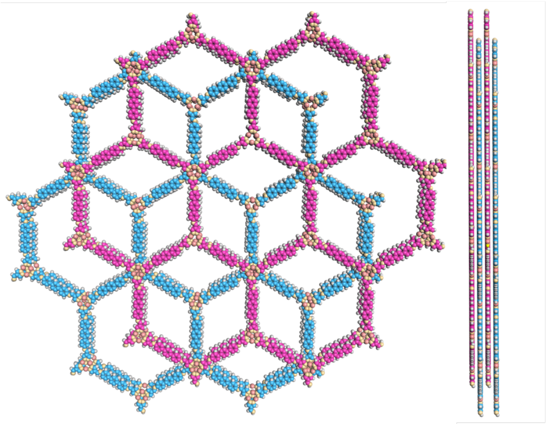

Supplementary Fig 6. Space-filling model of JNM-8-AB in AB stacking mode viewed from (left) *c* axis and (right) *a* axis.

Supplementary Table 3. Fractional atomic coordinates for JNM-8-AA.

JNM-8-AA

Space group symmetry: *P*6/*m*

$a = b = 49.3206$ Å; $c = 3.5288$ Å

$\alpha = \beta = 90.0000°$; $\gamma = 120.0000°$

Atom name	Atom	x	y	z
C1	C	0.4038	0.62449	0.51482
C2	C	0.37141	0.60777	0.51476
N3	N	1.2717	0.61459	0.51453
N4	N	1.26727	0.6389	0.51459
C5	C	1.24374	0.58808	0.51461
C6	C	1.18679	0.57471	0.51481
N7	N	0.72829	0.38543	0.52537
N8	N	0.73273	0.36113	0.52529
C9	C	0.76364	0.37145	0.52489
C10	C	0.7793	0.40384	0.52469
C11	C	0.75624	0.41194	0.52507
C12	C	0.81321	0.42534	0.52418
C13	C	0.8659	0.43252	0.51973
C14	C	0.88265	0.41749	0.42471
C15	C	0.91535	0.43392	0.42253
C16	C	0.93206	0.46581	0.52068
C17	C	0.91521	0.4808	0.62169
C18	C	0.88249	0.46438	0.62176
C19	C	0.96687	0.48334	0.51927
C20	C	0.9838	0.46877	0.63404
C21	C	1.0165	0.48524	0.63261
C22	C	1.03313	0.51671	0.51656
C23	C	1.01621	0.53128	0.40176
C24	C	0.9835	0.51481	0.40312
C25	C	1.08481	0.51929	0.41439
C26	C	1.06794	0.53425	0.51557
C27	C	1.08463	0.56613	0.61446
C28	C	1.11734	0.58256	0.61296
C29	C	1.1341	0.56755	0.51788
C30	C	1.11753	0.5357	0.41496
N31	N	1.16771	0.58567	0.52056
N32	N	0.83229	0.41439	0.51785
Cu33	Cu	0.31543	0.61888	0.5145
Cu34	Cu	0.69656	0.31545	0.52546
H35	H	0.35647	0.58259	0.51481
H36	H	1.24046	0.56469	0.51458
H37	H	1.17833	0.54983	0.51799
H38	H	0.77389	0.35652	0.52471
H39	H	0.7595	0.43533	0.52497
H40	H	0.82165	0.45022	0.52101
H41	H	0.87024	0.39296	0.34684

H42	H	0.92751	0.42173	0.33737
H43	H	0.9273	0.50509	0.70906
H44	H	0.87053	0.47663	0.71039
H45	H	0.97179	0.44481	0.73389
H46	H	1.02876	0.4735	0.73122
H47	H	1.02822	0.55525	0.30209
H48	H	0.97125	0.52656	0.30451
H49	H	1.07272	0.49501	0.3264
H50	H	1.07246	0.57829	0.69973
H51	H	1.12973	0.60707	0.69145
H52	H	1.1295	0.52348	0.3261

Supplementary Table 4. Fractional atomic coordinates for JNM-8-AB.

JNM-8-AB

Space group symmetry: *P*63/*m*

a = 48.6500 Å; b = 48.6500 Å; c = 6.7975 Å

$\alpha = \beta$ = 90.0000°; γ = 120.0000°

Atom name	Atom	x	y	z
C1	C	0.73189	1.28755	0.25
C2	C	0.69896	1.27121	0.25
N3	N	0.60623	1.28424	0.25
N4	N	0.6031	1.30962	0.25
C5	C	0.57757	1.25786	0.25
C6	C	0.52118	1.24748	0.25
N7	N	1.06043	1.0491	0.25
N8	N	1.06356	1.02371	0.25
C9	C	1.09442	1.03229	0.25
C10	C	1.11101	1.06522	0.25
C11	C	1.08909	1.07547	0.25
C12	C	1.14548	1.08585	0.25
C13	C	1.19799	1.09312	0.25
C14	C	1.21615	1.07818	0.25
C15	C	1.24925	1.09617	0.25
C16	C	1.26476	1.12945	0.25
C17	C	1.24643	1.14436	0.25
C18	C	1.21333	1.1264	0.25
C19	C	1.29986	1.1485	0.25
C20	C	1.3182	1.13361	0.25
C21	C	0.35129	1.15157	0.25
C22	C	0.3668	1.18483	0.25
C23	C	0.34847	1.19972	0.25
C24	C	1.31538	1.18176	0.25
C25	C	0.42024	1.18897	0.25
C26	C	0.4019	1.20388	0.25
C27	C	0.41742	1.23716	0.25

C28	C	0.45052	1.25515	0.25
C29	C	0.46868	1.24021	0.25
C30	C	0.45333	1.20694	0.25
N31	N	0.50254	1.25932	0.25
N32	N	1.16412	1.07401	0.25
Cu33	Cu	0.64777	1.28756	0.25
Cu34	Cu	1.02688	0.9811	0.25
H35	H	0.68268	1.24457	0.25
H36	H	0.57238	1.23245	0.25
H37	H	0.51057	1.22062	0.25
H38	H	1.10477	1.01601	0.25
H39	H	1.09428	1.10089	0.25
H40	H	1.15609	1.11271	0.25
H41	H	1.20388	1.05116	0.25
H42	H	1.26381	1.0837	0.25
H43	H	1.25854	1.17137	0.25
H44	H	1.19866	1.13875	0.25
H45	H	1.30609	1.1066	0.25
H46	H	0.36596	1.13921	0.25
H47	H	0.36057	1.22673	0.25
H48	H	1.30071	1.19412	0.25
H49	H	0.40813	1.16196	0.25
H50	H	0.40286	1.24964	0.25
H51	H	0.46279	1.28217	0.25
H52	H	0.468	1.19458	0.25

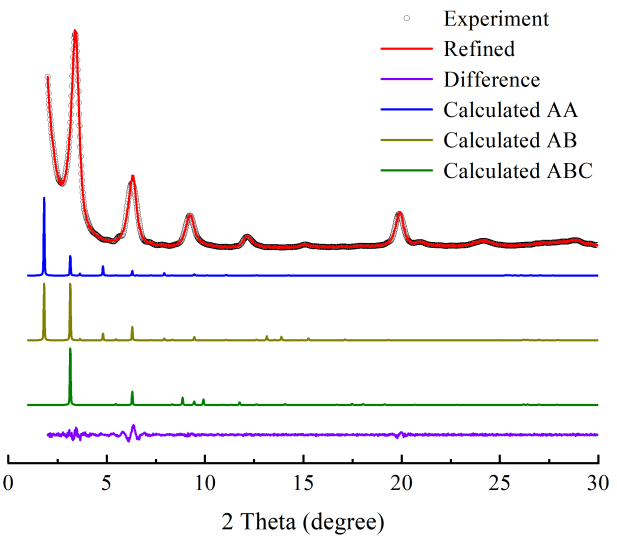

Supplementary Fig 7. PXRD patterns of JNM-9-ABC with the experimental profiles in red, difference curve in purple, and calculated profiles of AA (blue), AB (yellow), and ABC (green).

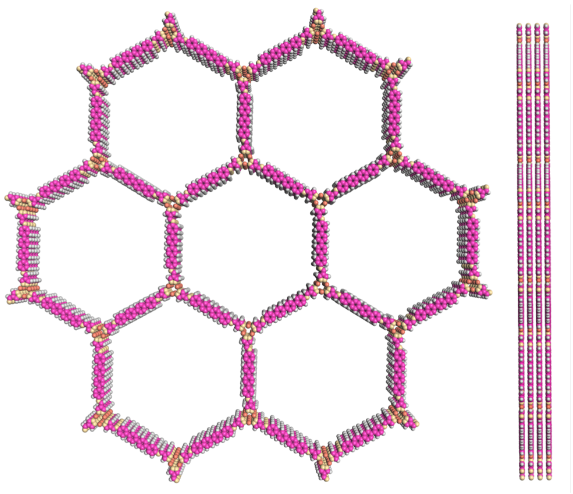

Supplementary Fig 8. Space-filling model of JNM-9-AA in AA stacking mode viewed from (left) *c* axis and (right) *a* axis.

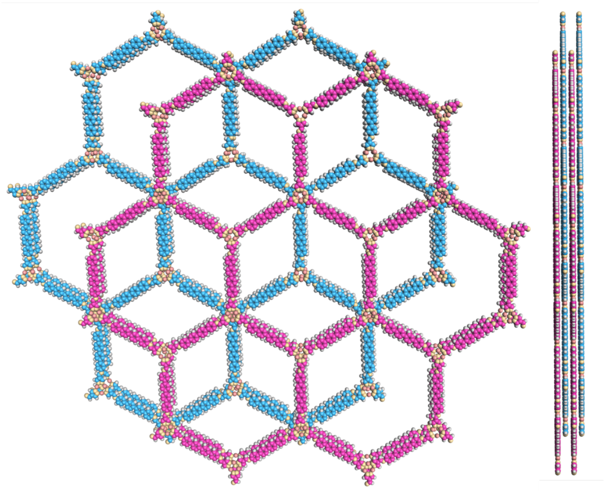

Supplementary Fig 9. Space-filling model of JNM-9-AB in AB stacking mode viewed from (left) *c* axis and (right) *a* axis.

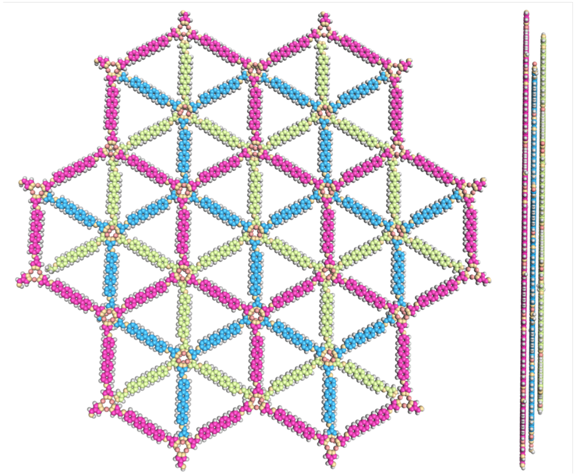

Supplementary Fig 10. Space-filling model of JNM-9-ABC in ABC stacking mode viewed from (left) *c* axis and (right) *a* axis.

Supplementary Table 5. Fractional atomic coordinates for JNM-9-AA.

JNM-9-AA

Space group symmetry: *P*6/*m*

$a = b = 56.1215$ Å; $c = 3.5215$ Å

$\alpha = \beta = 90.0000°$; $\gamma = 120.0000°$

Atom name	Atom	x	y	z
Cu1	Cu	0.37306	0.68976	-0.5
C2	C	0.38938	0.62638	-0.5
C3	C	0.40711	0.61419	-0.5
N4	N	0.62432	0.34352	-0.5
C5	C	0.46064	0.53393	-0.5
C6	C	0.44755	0.5051	-0.5
C7	C	0.44429	0.54803	-0.5
C8	C	0.39997	0.54606	-0.5
N9	N	0.39668	0.58774	-0.5
N10	N	0.27826	0.64644	-0.5
C11	C	-0.25587	0.39845	-0.5
C12	C	0.38763	0.74845	-0.5
C13	C	0.49181	1.02884	-0.5
C14	C	0.50705	1.01522	-0.5
C15	C	0.53588	1.03097	-0.5
C16	C	0.54917	1.05967	-0.5
C17	C	0.42313	0.88051	-0.5
C18	C	0.40984	0.8518	-0.5
C19	C	0.42523	0.83833	-0.5
C20	C	0.46722	0.88263	-0.5
H21	H	0.58931	0.24271	0.5
H22	H	0.49231	0.06818	0.5
H23	H	0.46838	0.01636	0.5
H24	H	0.54867	0.02034	0.5
H25	H	0.5726	0.07226	0.5
H26	H	0.50934	0.10499	0.5
H27	H	0.53346	0.15692	0.5
H28	H	0.61359	0.16074	0.5
H29	H	0.58956	0.10875	0.5
H30	H	0.43044	0.62802	0.5
H31	H	0.32825	0.74878	0.5

Supplementary Table 6. Fractional atomic coordinates for JNM-9-AB.

JNM-9-AB

Space group symmetry: *P*63/*m*

$a = b = 56.1215$ Å; $c = 6.8003$ Å

$\alpha = \beta = 90.0000°$; $\gamma = 120.0000°$

Atom name	Atom	x	y	z
Cu1	Cu	-0.29361	0.35643	0.25
C2	C	-0.27729	0.29305	0.25
C3	C	-0.25955	0.28086	0.25
N4	N	-0.04234	0.01019	0.25
C5	C	-0.20603	0.2006	0.25
C6	C	-0.21911	0.17177	0.25
C7	C	-0.22238	0.2147	0.25
C8	C	-0.26669	0.21272	0.25
N9	N	-0.26998	0.2544	0.25
N10	N	-0.3884	0.31311	0.25
C11	C	0.09633	0.05605	0.25
C12	C	0.07747	0.06512	0.25
C13	C	0.12625	0.07378	0.25
C14	C	-0.27904	0.41512	0.25
N15	N	-0.32314	0.38587	0.25
C16	C	-0.32098	0.4108	0.25
C17	C	-0.05429	-0.08178	0.25
N18	N	-0.03484	-0.05507	0.25
C19	C	-0.13274	-0.26004	0.25
C20	C	-0.16156	-0.27578	0.25
C21	C	-0.17485	-0.30449	0.25
C22	C	-0.15962	-0.31811	0.25
C23	C	-0.13079	-0.30237	0.25
C24	C	-0.1175	-0.27366	0.25
C25	C	-0.17371	0.65144	0.25
C26	C	-0.20254	0.6357	0.25
C27	C	-0.15848	0.63782	0.25
C28	C	-0.21583	0.60699	0.25
C29	C	-0.24354	0.54718	0.25
C30	C	-0.25682	0.51847	0.25
C31	C	-0.24143	0.505	0.25
C32	C	-0.19945	0.5493	0.25
C33	C	-0.11864	-0.22959	0.25
C34	C	-0.13389	-0.21597	0.25
C35	C	-0.12061	-0.18725	0.25
C36	C	-0.0919	-0.17166	0.25
C37	C	-0.07651	-0.18514	0.25
C38	C	-0.0898	-0.21385	0.25
N39	N	-0.07893	-0.14228	0.25
Cu40	Cu	0.01663	0.03972	0.25
H41	H	-0.23623	0.29468	0.25

H42	H	-0.24253	0.15898	0.25
H43	H	-0.29013	0.20012	0.25
H44	H	0.08212	0.0872	0.25
H45	H	0.13575	0.0971	0.25
H46	H	-0.25597	0.42395	0.25
H47	H	-0.33841	0.41545	0.25
H48	H	-0.07736	-0.09062	0.25
H49	H	-0.17435	-0.26515	0.25
H50	H	-0.19829	-0.31697	0.25
H51	H	-0.118	-0.313	0.25
H52	H	-0.09407	-0.26108	0.25
H53	H	-0.21533	0.64633	0.25
H54	H	-0.13504	0.6503	0.25
H55	H	-0.23927	0.59441	0.25
H56	H	-0.25623	0.55792	0.25
H57	H	-0.28026	0.50593	0.25
H58	H	-0.176	0.56168	0.25
H59	H	-0.15733	-0.22835	0.25
H60	H	-0.13321	-0.17642	0.25
H61	H	-0.05307	-0.17259	0.25
H62	H	-0.07711	-0.22459	0.25

Supplementary Table 7. Fractional atomic coordinates for JNM-9-ABC.

JNM-9-ABC

Space group symmetry: *R*-3

a = *b* = 56.1215 Å; *c* = 10.2004 Å

α = *β* = 90.0000°; *γ* = 120.0000°

Atom name	Atom	x	y	z
Cu1	Cu	-0.37306	-0.68976	0.00001
C2	C	-0.38938	-0.62638	0.00001
C3	C	-0.40711	-0.61419	0
N4	N	-0.62432	-0.34352	-0.00001
C5	C	-0.46064	-0.53393	0
C6	C	-0.44755	-0.5051	0
C7	C	-0.44429	-0.54803	0
C8	C	-0.39997	-0.54606	0
N9	N	-0.39668	-0.58774	0
N10	N	-0.27826	-0.64644	0.00001
C11	C	-0.74413	-0.39845	-0.00001
C12	C	-0.38763	-0.74845	0.00001
C13	C	-0.49181	-0.02884	0
C14	C	-0.50705	-0.01522	0
C15	C	-0.53588	-0.03097	0
C16	C	-0.54917	-0.05967	0
C17	C	-0.42313	-0.88051	0
C18	C	-0.40984	-0.8518	0
C19	C	-0.42523	-0.83833	0
C20	C	-0.46722	-0.88263	0
H21	H	-0.43044	-0.62802	0
H22	H	-0.42413	-0.49231	0
H23	H	-0.37654	-0.53346	0
H24	H	-0.74878	-0.42053	-0.00001
H25	H	-0.41069	-0.75729	0.00001
H26	H	-0.46838	-0.01636	0
H27	H	-0.54867	-0.02034	0
H28	H	-0.5726	-0.07226	0
H29	H	-0.41044	-0.89125	0
H30	H	-0.38641	-0.83926	0
H31	H	-0.49066	-0.89501	0

3.2 Energy Dispersive X-ray Spectroscopy (EDS)

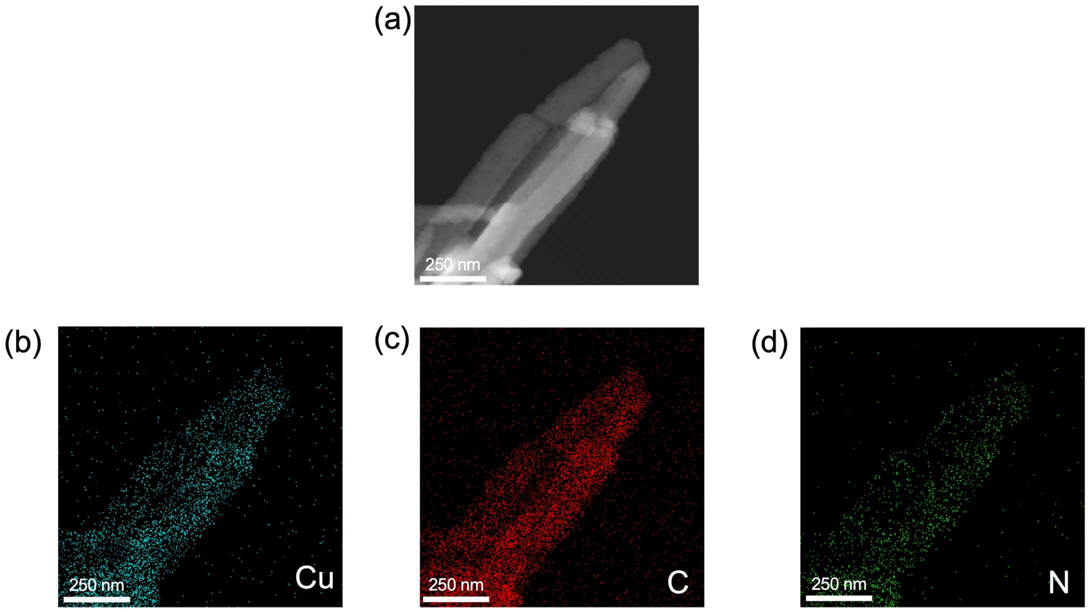

Supplementary Fig 11. EDS of **JNM-7-AA**. (a) TEM photo of **JNM-7-AA** (scale bar, 250 nm); Element mapping of (b) Cu: Blue; (c) C: Red; (d) N: Green.

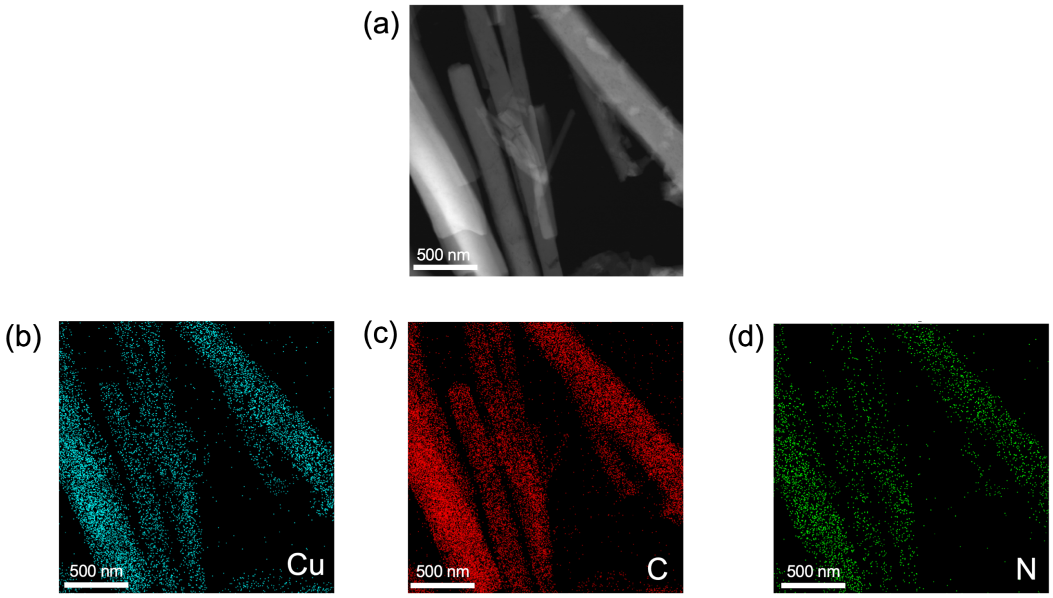

Supplementary Fig 12. EDS of **JNM-8-AA**. (a) TEM photo of **JNM-8-AA** (scale bar, 500 nm); Element mapping of (b) Cu: Blue; (c) C: Red; (d) N: Green.

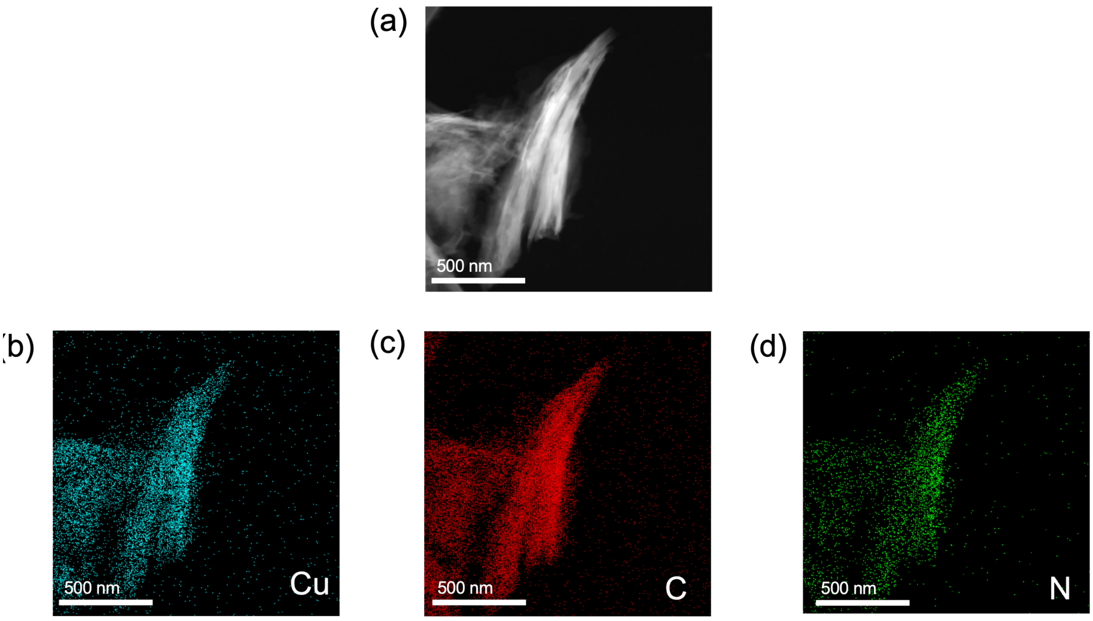

Supplementary Fig 13. EDS of **JNM-9-ABC**. (a) TEM photo of **JNM-9-ABC** (scale bar, 500 nm); Element mapping of (b) Cu: Blue; (c) C: Red; (d) N: Green.

3.3 Gas adsorption isotherms and pore size distribution of JNM-9-ABC.

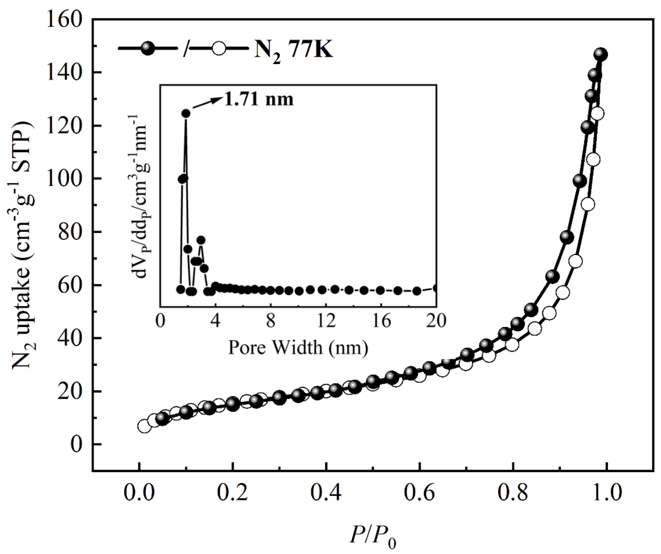

Supplementary Fig 14. N_2 adsorption-desorption isotherms and pore size distribution profiles of JNM-9-ABC.

4. Reversible Structure transformation

4.1 Experimental procedures

Structure transformation from AA to ABC. To a 10 mL Schlenk tube, pristine sample of JNM-7-AA (50 mg) or JNM-8-AA (50 mg) and 5 mL DMF was added. The resulting mixture was stirred at 80 °C for 10 h, and then the solid was isolated by filtration, washed and solvent exchanged with EtOH and acetone several times. The resulting powder was dried under vacuum at room temperature for 3 h. Finally, the pale-yellow powders JNM-7-ABC or JNM-8-ABC were obtained. JNM-7-ABC IR (KBr): ν = 1669 (m), 1624 (s), 1594 (m), 1543 (m), 1489 (s), 1406 (w), 1376 (w), 1306 (w), 1241 (m), 1202 (s), 1241 (m), 1056cm^{-1} (m). JNM-8-ABC IR (KBr): ν = 1669 (m), 1625 (s), 1589 (m), 1532 (m), 1484 (s), 1404 (w), 1376 (w), 1320 (w), 1201 (s), 1175 (w), 1056 cm^{-1} (m).

Structure transformation from ABC to AA. JNM-7-ABC (20 mg) or JNM-8-ABC (20 mg), 0.5 mL of 1,2-dichlorobenzene, 0.5 mL of 1-butanol, and 0.1 mL of TFA (6 M) were added a 10 mL Schlenk tube. The resulting mixture was stirred at 80 °C for 10 h, and then the solid was isolated by filtration, washed and solvent exchanged with EtOH and acetone for several times. The resulting powder was dried under vacuum at room temperature for 3 h. Finally, the brown powder JNM-7-AA or JNM-8-AA with similar diffraction peaks to the AA simulation was obtained.

4.2 Structural simulation for JNM-7-ABC and JNM-8-ABC

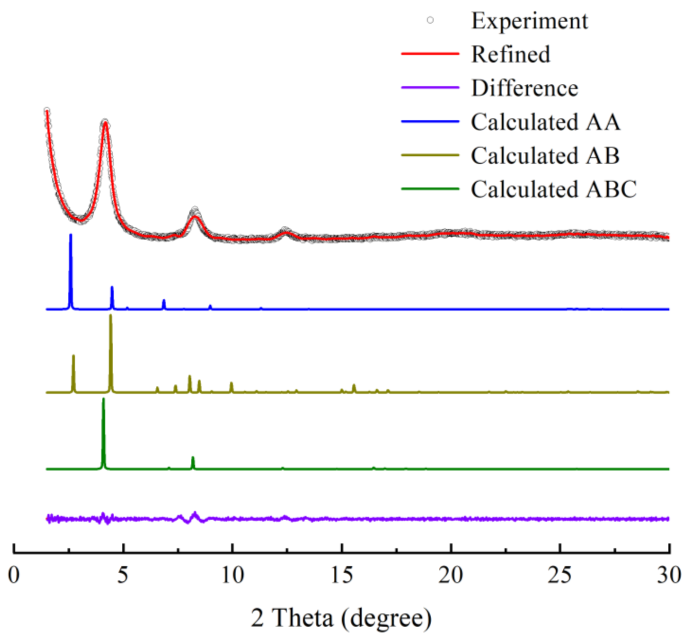

Supplementary Fig 15. PXRD patterns of **JNM-7-ABC** with the experimental profiles in red, difference curve in purple, and calculated profiles of AA (blue), AB (yellow), and ABC (green).

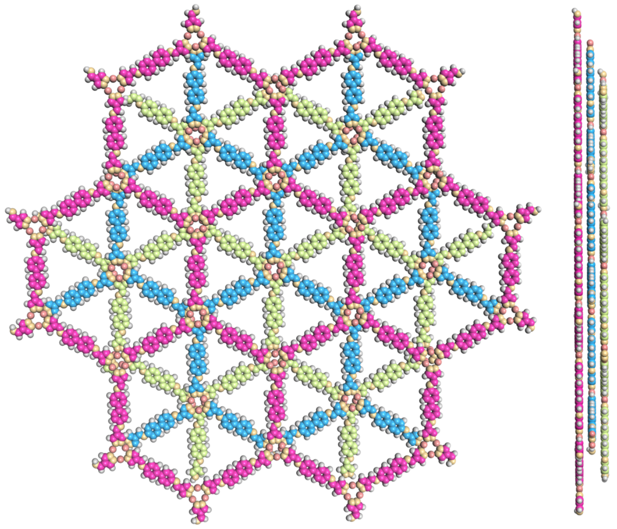

Supplementary Fig 16. Space-filling model of JNM-7-ABC in ABC stacking mode viewed from (left) *c* axis and (right) *a* axis.

Supplementary Table 8. Fractional atomic coordinates for JNM-7-ABC.

JNM-7-ABC

Space group symmetry: *R*-3

$a = b = 41.1875$ Å; $c = 5.6230$ Å; $\alpha = \beta = 90.0000°$; $\gamma = 120.0000°$

Atom name	Atom	x	y	z
N1	N	-0.37755	-0.74196	0.25247
C2	C	-0.41528	-0.61789	0.24622
C3	C	-0.37653	-0.59776	0.24831
C4	C	-0.4409	-0.60307	0.23592
C5	C	-0.58443	-0.38177	0.09055
C6	C	-0.62318	-0.402	0.08756
N7	N	-0.63541	-0.37739	0.08371
C8	C	-0.60356	-0.16225	0.10167
C9	C	-0.44933	-0.54885	0.20225
C10	C	-0.48693	-0.56597	0.27555
C11	C	-0.50661	-0.54666	0.25921
C12	C	-0.48913	-0.50989	0.17126
C13	C	-0.45146	-0.49274	0.10155
C14	C	-0.4318	-0.51205	0.11797
C15	C	-0.51006	-0.48943	0.15344
C16	C	-0.54715	-0.5076	0.07105
C17	C	-0.56684	-0.48831	0.05483
C18	C	-0.5499	-0.45056	0.11849
C19	C	-0.51288	-0.43236	0.20369
C20	C	-0.49317	-0.45163	0.21995
N21	N	-0.42774	-0.56732	0.21546
N22	N	-0.57167	-0.43228	0.10217
Cu23	Cu	-0.31329	-0.61297	0.25288
N24	N	-0.26336	-0.60667	0.25321
C25	C	-0.23014	-0.57492	0.25
N26	N	-0.73656	-0.39345	0.08372
C27	C	-0.76972	-0.42525	0.08762
Cu28	Cu	-0.68657	-0.387	0.08367
H29	H	-0.35864	-0.56771	0.24532
H30	H	-0.47063	-0.62261	0.2374
H31	H	-0.64099	-0.43206	0.09036
H32	H	-0.62323	-0.15222	0.10609
H33	H	-0.50113	-0.59387	0.34925
H34	H	-0.53537	-0.56029	0.31919
H35	H	-0.43731	-0.46454	0.03137
H36	H	-0.40282	-0.49841	0.06251
H37	H	-0.56082	-0.53662	0.01666
H38	H	-0.5954	-0.50278	-0.00912
H39	H	-0.49921	-0.40361	0.26259
H40	H	-0.4649	-0.43714	0.28891
H41	H	-0.22635	-0.54705	0.24797
H42	H	-0.77342	-0.45309	0.09035

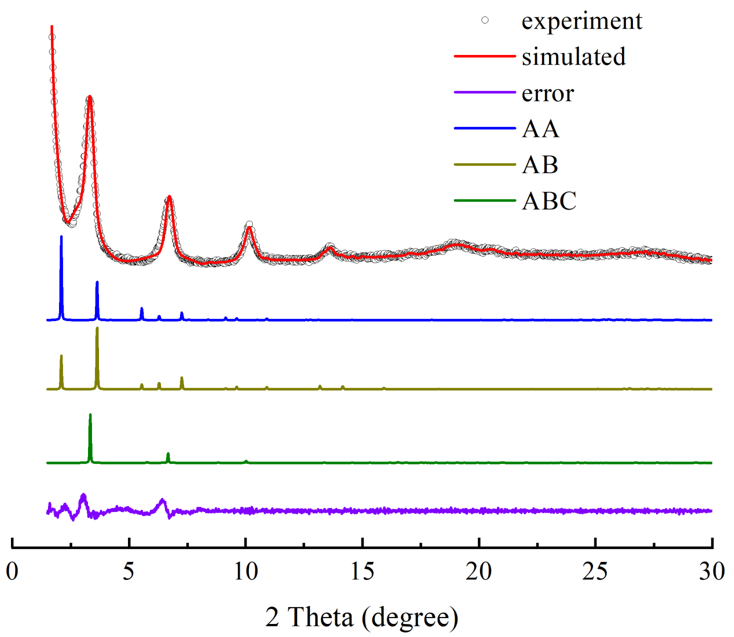

Supplementary Fig 17. PXRD patterns of **JNM-8-ABC** with the experimental profiles in red, difference curve in purple, and calculated profiles of AA (blue), AB (yellow), and ABC (green).

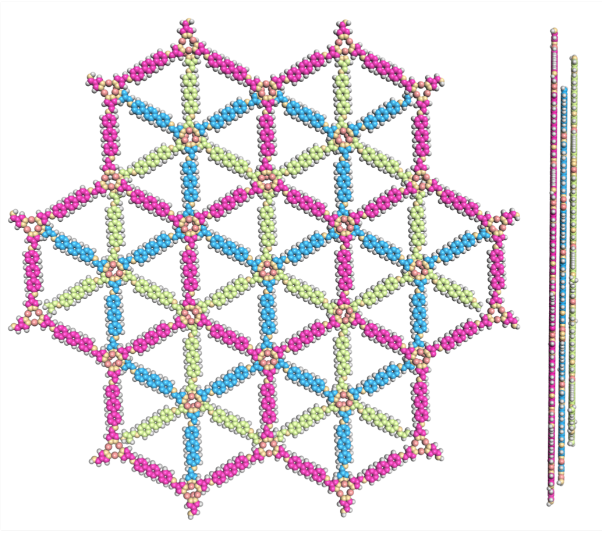

Supplementary Fig 18. Space-filling model of JNM-8-ABC in ABC stacking mode viewed from (left) *c* axis and (right) *a* axis.

Supplementary Table 9. Fractional atomic coordinates for JNM-8-ABC.

JNM-8-ABC

Space group symmetry: *R*-3

$a = b = 48.6710$ Å; $c = 10.2001$ Å

$\alpha = \beta = 90.0000°$; $\gamma = 120.0000°$

Atom name	Atom	x	y	z
C1	C	-0.39856	-0.62088	0
C2	C	-0.36563	-0.60454	0
N3	N	-0.2729	-0.61757	0
N4	N	-0.26977	-0.64295	0
C5	C	-0.24424	-0.59119	0
C6	C	-0.18785	-0.58081	0
C7	C	-0.86466	-0.42645	0
C8	C	-0.88281	-0.41152	0
C9	C	-0.91591	-0.4295	0
C10	C	-0.93143	-0.46279	0
C11	C	-0.91309	-0.47769	0
C12	C	-0.88	-0.45973	0
C13	C	-0.96653	-0.48184	0
C14	C	-0.98487	-0.46694	0
C15	C	-0.01796	-0.4849	0
N16	N	-0.16921	-0.59266	0
Cu17	Cu	-0.31444	-0.62089	0
H18	H	-0.34936	-0.57792	0
H19	H	-0.23905	-0.56579	0
H20	H	-0.17724	-0.55397	0
H21	H	-0.87055	-0.38451	0
H22	H	-0.93047	-0.41704	0
H23	H	-0.9252	-0.50469	0
H24	H	-0.86534	-0.47208	0
H25	H	-0.97277	-0.43995	0
H26	H	-0.03262	-0.47255	0

4.3 Reversible structure transformation monitoring by PXRD.

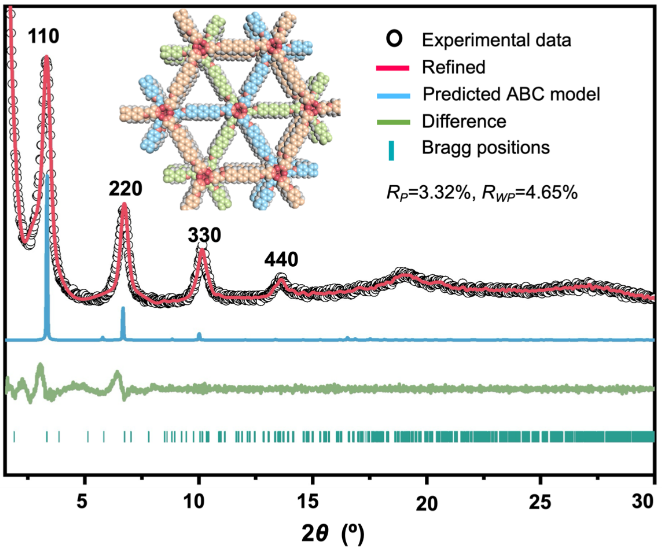

Supplementary Fig 19. PXRD structural analysis of JNM-8-ABC showing space-filling model (C, light pink; H, white; Cu, red; N, pink).

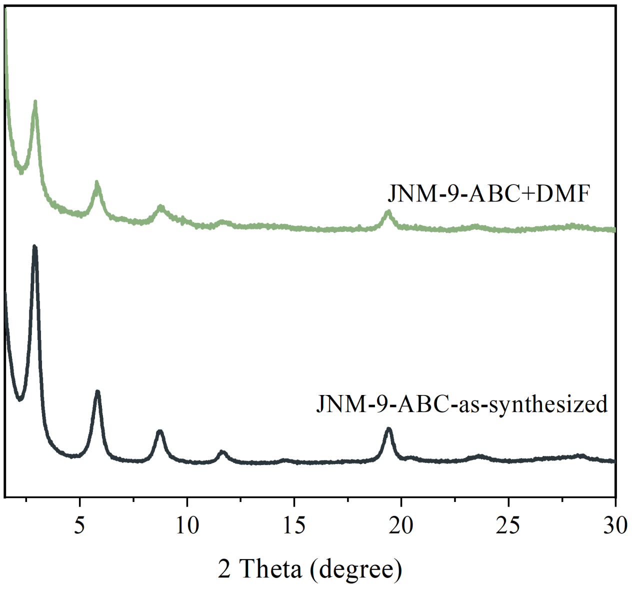

Supplementary Fig 20. PXRD patterns of JNM-9-ABC into DMF solution at 80 °C for 10 h.

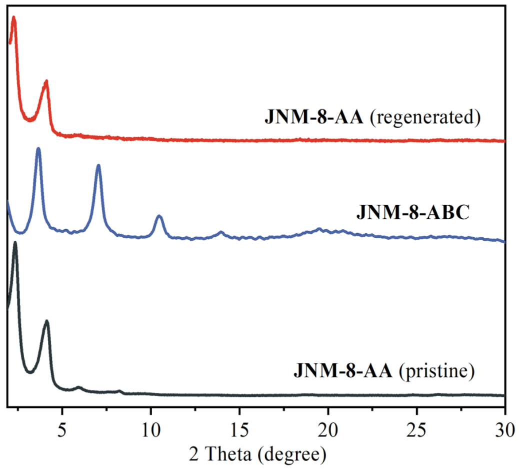

Supplementary Fig 21. Reversible structure transformation of JNM-8-AA (pristine), JNM-8-ABC and JNM-8-AA (regenerated) monitoring by PXRD analysis.

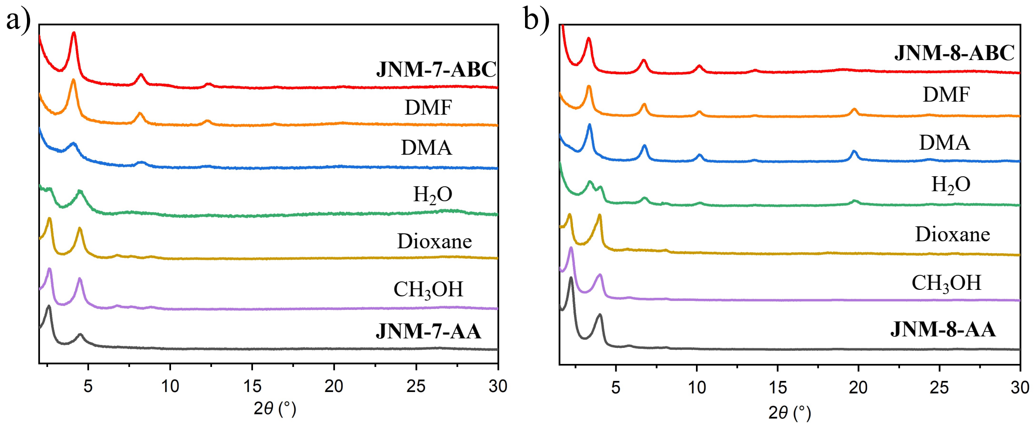

Supplementary Fig 22. PXRD analysis of (a) JNM-7-AA and (b) JNM-8-AA after treatment with various solvent at 80 °C for 8 h.

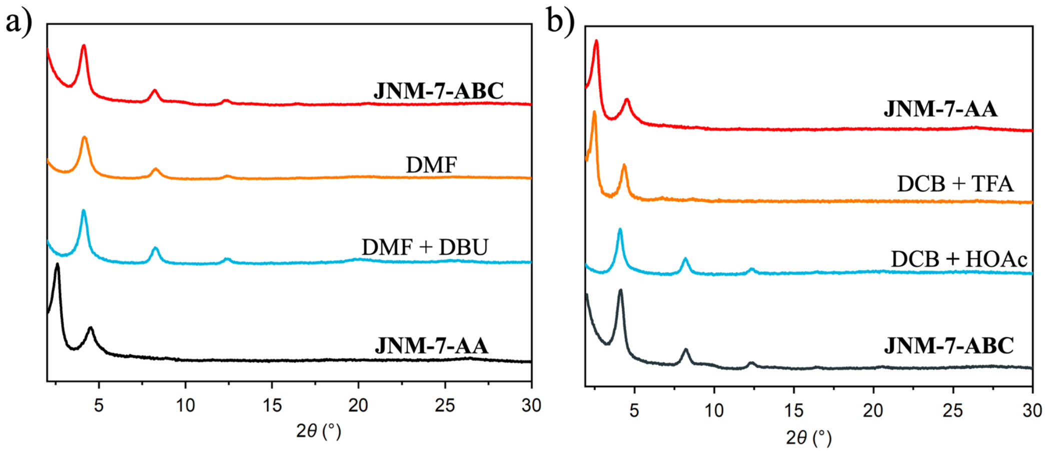

Supplementary Fig 23. PXRD analysis of JNM-7-AA after treatment with a mixture of (a) DMF and various base (i.e., DBU and ammonia (aq.)) and (b) DCB with acid (i.e., TFA and HOAc) at 80 °C for 8 h.

4.4 Fourier-transform infrared (FT-IR) spectra

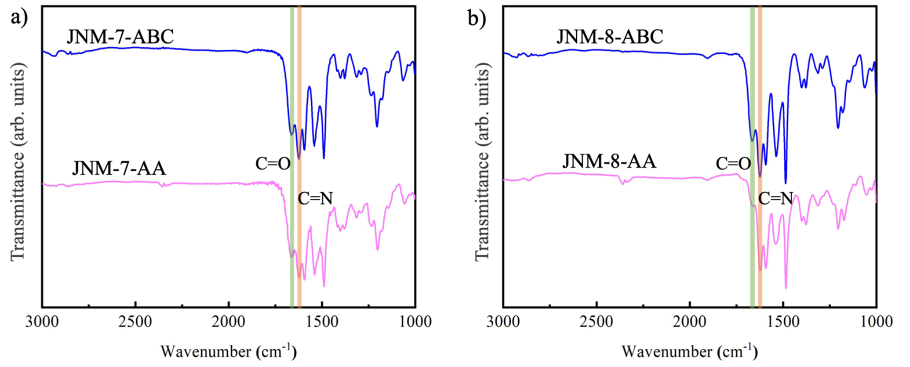

Supplementary Fig 24. FT-IR analyses of (a) JNM-7 and (b) JNM-8.

4.5 Solid-state ^{13}C CP/MAS NMR spectra.

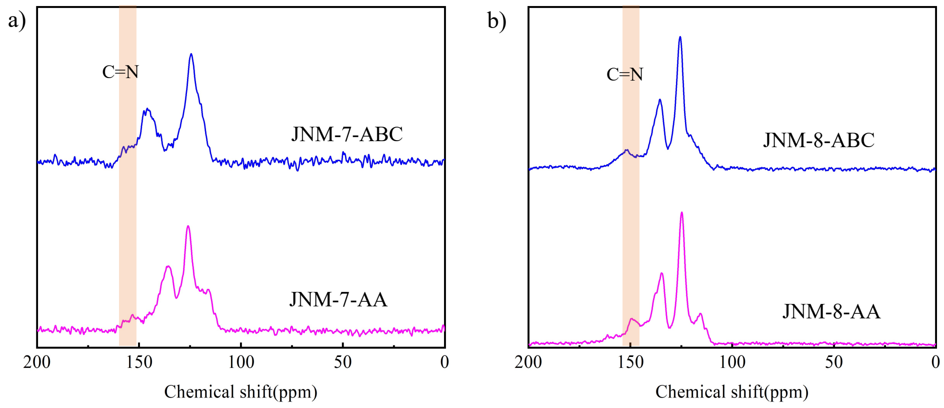

Supplementary Fig 25. Solid-state ^{13}C CP/MAS NMR spectra and peak assignments for (a) **JNM-7 and (b) JNM-8.**

4.6 Scanning electron microscopy (SEM).

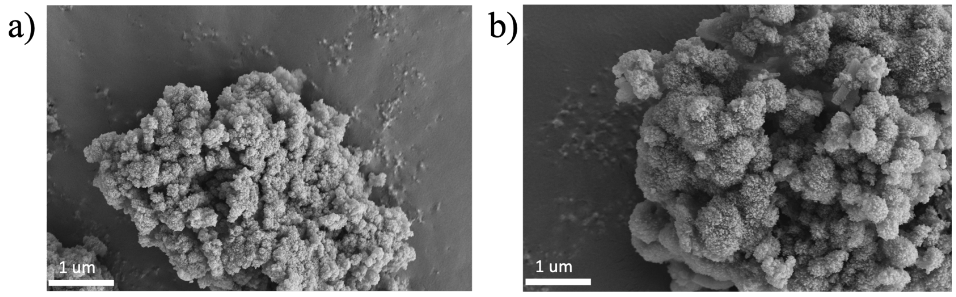

Supplementary Fig 26. SEM analyses of (a) JNM-7-ABC and (b) JNM-8-ABC**.**

4.7 X-ray photoelectron spectroscopy (XPS).

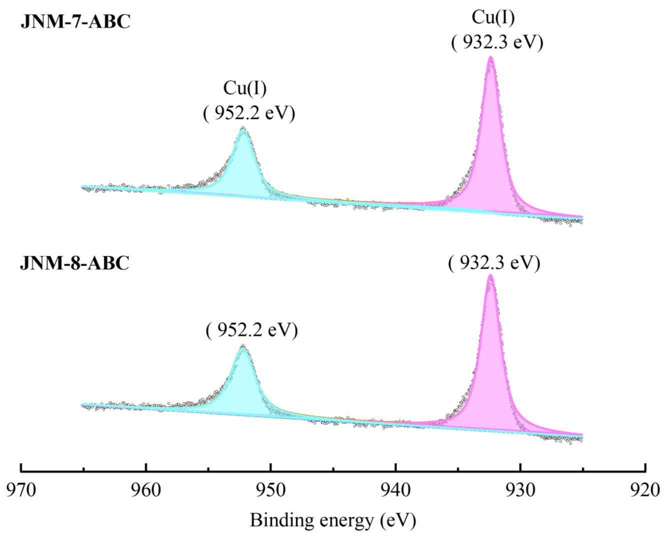

Supplementary Fig 27. XPS spectra of JNM-7-ABC and JNM-8-ABC.

4.8 Thermogravimetric analysis (TGA)

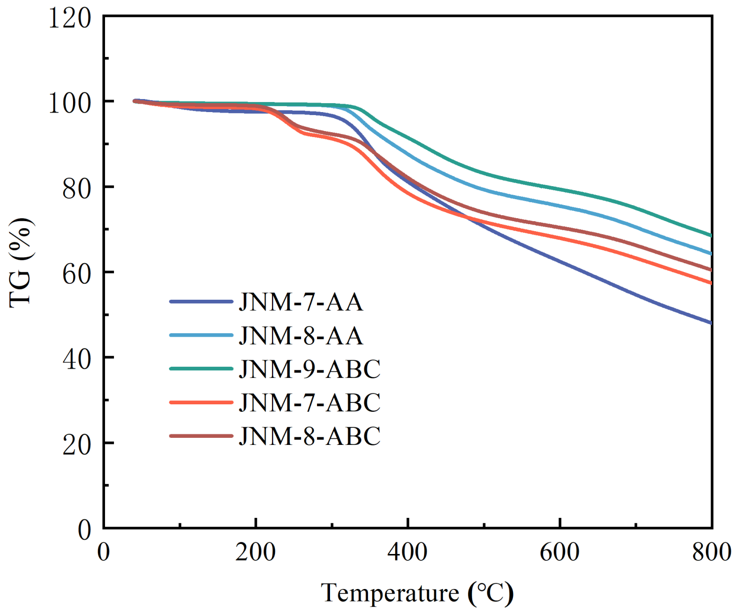

Supplementary Fig 28. TGA curve of **JNM-7-AA, JNM-8-AA, JNM-9-ABC, JNM-7-ABC, and JNM-8-ABC** under N_2 atmosphere.

4.9 Various-Temperature PXRD.

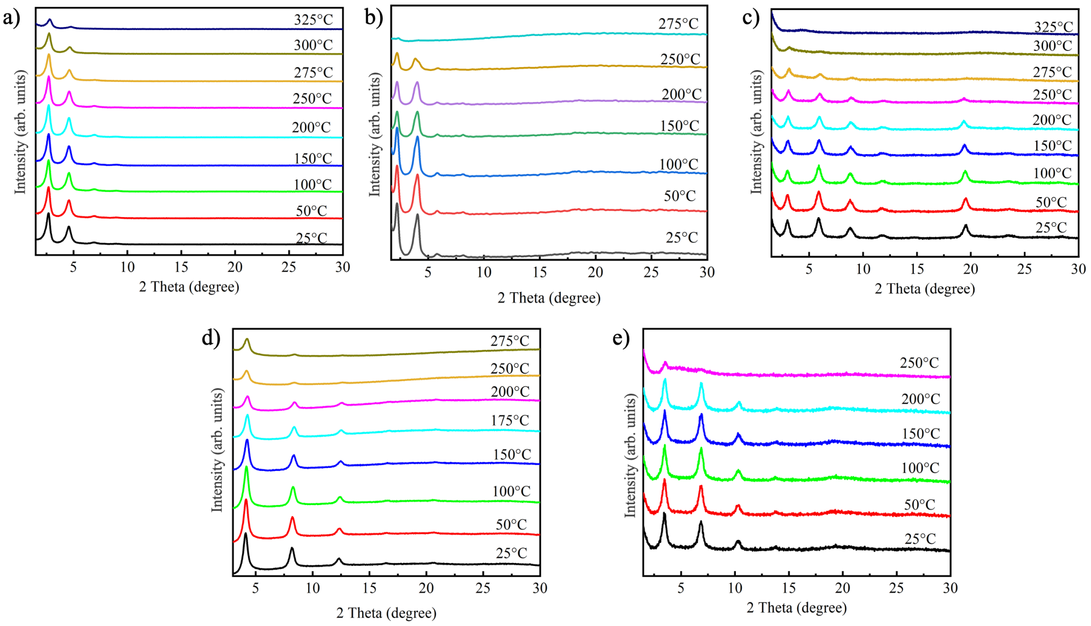

Supplementary Fig 29. PXRD patterns for samples of (a) **JNM-7-AA, (b) JNM-8-AA, (c) JNM-9-ABC,** (d) **JNM-7-ABC and (e) JNM-8-ABC** form 25°C to 300°C.

4.10 Stability in various solvents.

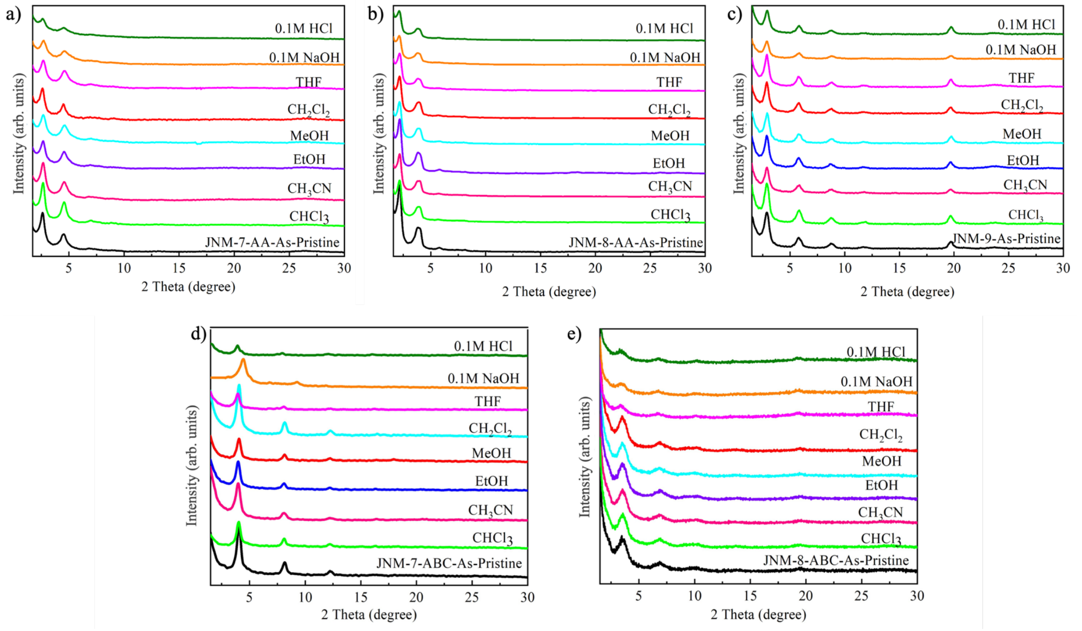

Supplementary Fig 30. PXRD patterns for samples of (a) **JNM-7-AA, (b) JNM-8-AA, (c) JNM-9-ABC,** (d) **JNM-7-ABC and (e)JNM-8-ABC** after treatment with different solvents for 24 h.

4.11 Gas adsorption isotherms and pore size distribution of JNM-8.

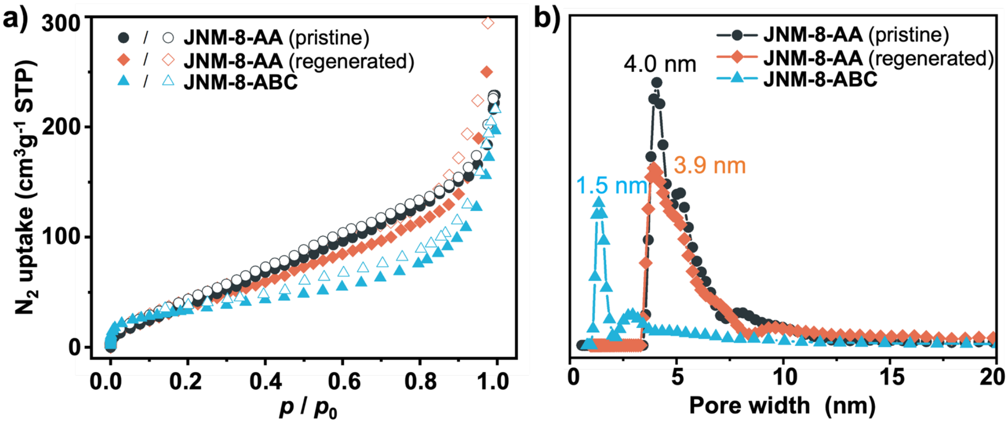

Supplementary Fig 31. Reversible structure transformation (a) BET surface analysis and (b) pore size distribution profiles of JNM-8-AA (pristine), JNM-8-ABC and JNM-8-AA (regenerated).

4.12 DFT calculations[2, 3]

All calculations were performed by using the density functional method conducted out by the DMol^3 molecular dynamics module embedded in Materials Studio 2018 (MS 2018). Generalized gradient approximation (GGA) and Perdew–Burke–Ernzerhof (PBE) were used. The total energy difference and maximum residual force converged within 10^{-4} Ha and 0.05 Ha/Å during optimization. In all calculations, we used periodic boundary conditions and a supercell large enough to present the full coordination environments of JNM-7. The structures of the simulated AA stacked and ABC stacked JNM-7 were firstly optimized, then solvent molecules were introduced to the channel pore.

Supplementary Table 10. The cell parameters, total energy of the possible structures of JNM-7 under different states.

Structure	Formula	*c* (Å)	Total energy (kcal/mol)
JNM-7-AA	$C_{180}H_{126}N_{54}Cu_{18}$	10.25	-8713563.47
JNM-7-ABC	$C_{180}H_{126}N_{54}Cu_{18}$	10.25	-8713373.43
JNM-7-AA-DMF	$C_{183}H_{133}N_{55}OCu_{18}$	10.25	-8869306.91
JNM-7-ABC-DMF	$C_{183}H_{133}N_{55}OCu_{18}$	10.25	-8869097.28
NM-7-AA-TFA	$C_{182}H_{127}N_{54}Cu_{18}O_2F_3$	10.25	-9043675.61
JNM-7-ABC-TFA	$C_{182}H_{127}N_{54}Cu_{18}O_2F_3$	10.25	-9043841.94
DMF	C_3H_7NO	10.25	-155779.57
TFA	$C_2HO_2F_3$	10.25	-330372.34

Supplementary Table 11. The space group, cell parameters, total energy, and total crystal stacking energy per layer of the possible structures of JNM-9 under different states.

Structure	Space group	Formula	*c* (Å)	Total energy (kcal/mol)	Total crystal stacking energy per layer (kcal/mol)[a]
Monolayer				-3773315.589	
JNM-9-AA	*P*6	$C_{96}H_{66}N_{18}Cu_6$	3.5215	-3773421.396	105.8065731
JNM-9-AB	*P*63/*m*	$C_{192}H_{132}N_{36}Cu_{12}$	6.8003	-7546709.441	39.13155517
JNM-9-ABC	*R*-3	$C_{288}H_{198}N_{54}Cu_{18}$	10.2004	-11320129.62	60.95219402

[a] $\Delta E_{AA/layer} = E_{monolayer} - E_{AA}$, $\Delta E_{AB/layer} = (E_{monolayer} \times 2 - E_{AB})/2$, $\Delta E_{ABC/layer} = (E_{monolayer} \times 3 - E_{ABC})/3$

4.13 Reversible encapsulation and release of lipase

Encapsulation of lipase. In a typical adsorption experiment, 15 mg of JNM-8-AA was added to 1 mL of an aqueous phosphate buffer solution of lipase (pH = 7.0, C_0 = 30 mg/mL), and the mixture was shaken at 175 rpm and 25 °C for 6 h. Afterward, the resultants were centrifuged and the solid was collected to give LP@JNM-8-AA. The supernatant was further analyzed by UV-Vis spectroscopy using the *n*-butyl cyanoacrylate (BCA) method to provide give the concentration of lipase (C_{at}).

The adsorption efficiency of lipase was calculated as following equation (1):

Adsorption efficiency (%) = $(C_0 - C_{at}) / C_0 \times 100\%$ (1)

C_0 and C_{at} are the lipase concentrations concentration of lipase at the initial condition and in the filtrate after adsorption, respectively.

Release of lipase. The LP@JNM-8-AA obtained in the above adsorption process was added to an aqueous phosphate buffer (0.5 mL), and DMF mixture solution (0.5 mL), and then the mixture was heated at 80 °C for 6h, followed by cooling down to room temperature. Afterward, the resultants were centrifuged and filtrated, and the solid was collected to give JNM-8-AB**C**. The filtrate was further analyzed by UV-Vis spectroscopy using the *n*-butyl cyanoacrylate (BCA) method to provide the concentration of lipase (C_{dt}).

The desorption efficiency of lipase was calculated as following equation (2):

$$\text{Desorption efficiency (\%)} = C_{dt} / (C_0 - C_{at}) \times 100 \quad (2)$$

C_0, $C_{at,}$ and C_{dt} are the lipase concentration at the initial condition, in the filtrate after the adsorption process, and in the filtrate after the desorption process, respectively.

Preparation of the BCA Working Reagent[4, 5]:

The BCA working reagent was first prepared by mixing 50 parts of BCA Reagent A with 1 part of BCA Reagent B (Cu^{2+} solution). As a typical run, combine 5 mL of Reagent A with 100 μL of Reagent B.

Test-tube Procedure:

I: Pipette 140 μL of each standard and unknown sample into an appropriately labeled 10mL test tube.

II: Add 2.8 mL of the working reagent to each tube and mix well.

III: Cover tubes at 55 ºC for 15 min.

IV: Cool all tubes to RT for 30 min.

V: Prepare a standard curve. Use the standard curve to determine the protein concentration of each unknown sample.

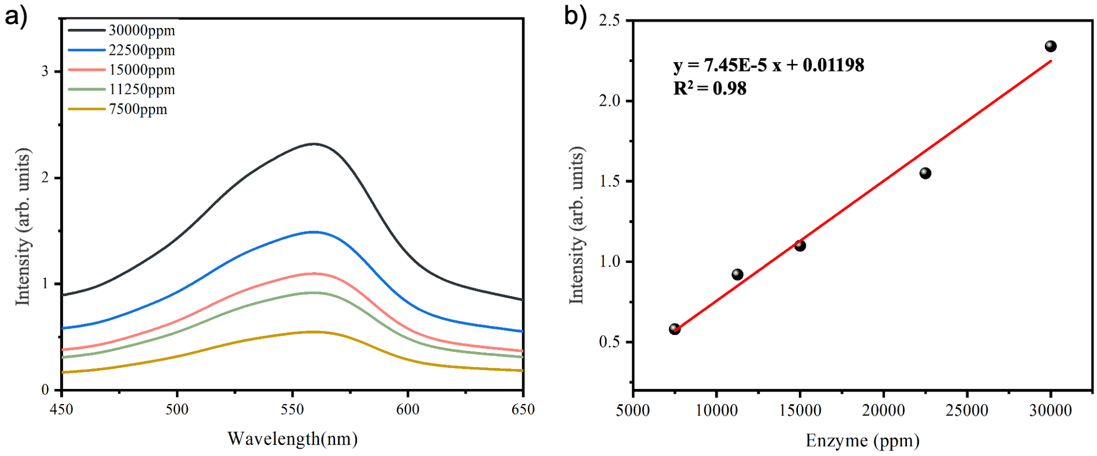

Supplementary Fig 32. (a) UV-Vis spectra of lipase with known concentrations for preparing (b) standard curve.

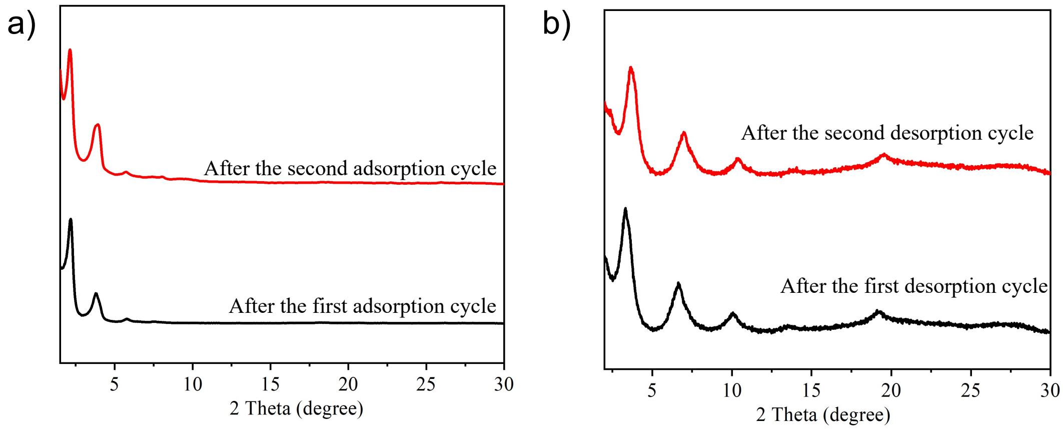

Supplementary Fig 33. PXRD patterns for (a) JNM-8-AA (b) JNM-8-ABC after lipase adsorption and desorption twice.

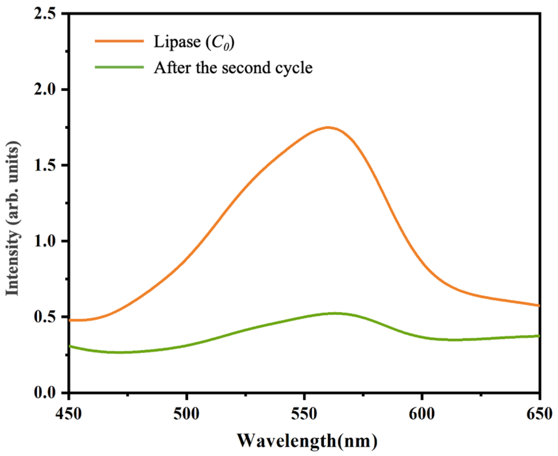

Supplementary Fig 34. UV-Vis spectrum of residual lipase concentrations after adsorption and desorption of JNM-8 in the second cycle

5. Optical and electronic properties

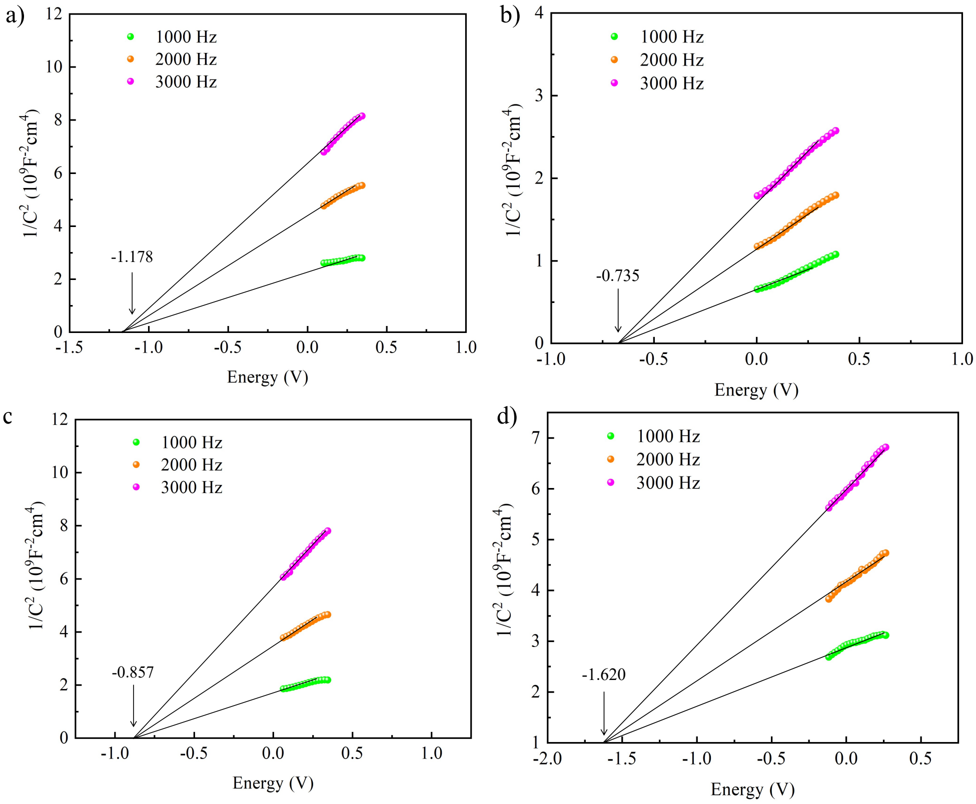

Supplementary Fig 35. Mott-Schottky plots for (a) JNM-7-AA, (b) JNM-7-ABC, (c) JNM-8-AA and (d) JNM-8-ABC in 0.1 M Na_2SO_4 aqueous solution.

Supplementary Table 12. The EIS simulated resistance values of JNMs.

Sample	R_s (Ω)	R_{ct} (Ω)
JNM-7-AA	17.57	39.74
JNM-7-ABC	23.41	75.30
JNM-8-AA	20.48	36.91
JNM-8-ABC	12.22	57.66

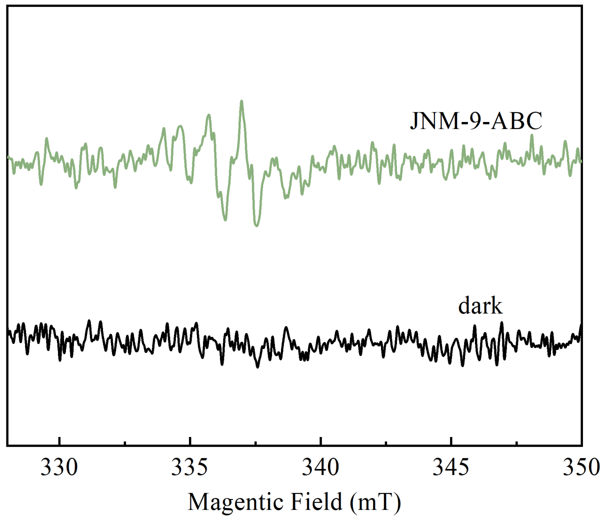

Supplementary Fig 36. Electron paramagnetic resonance (EPR) measures signals of $^1O_2$ for JNM-9-ABC**.**

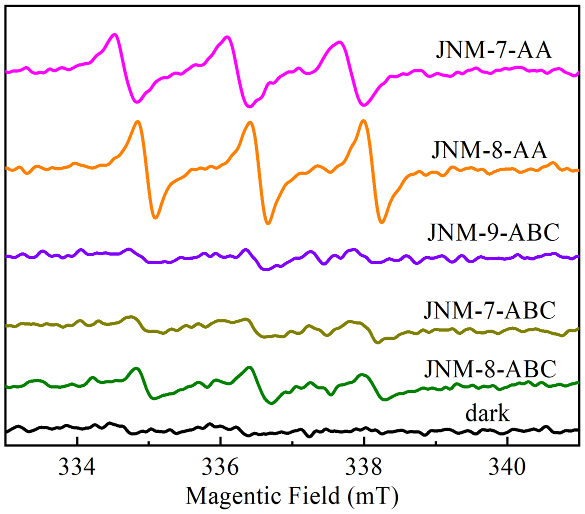

Supplementary Fig 37. Electron paramagnetic resonance (EPR) measures signals of $^1O_2$ for JNMs.

6. Photocatalytic Studies

6.1 Experiments of CDC reactions.

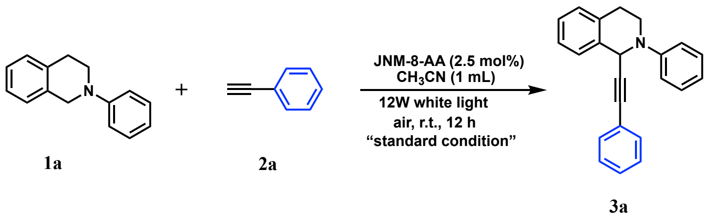

Before the catalytic experiment, the catalysts were dried in a vacuum at 120°C for 8h. 2-phenyl-1,2,3,4-tetrahydrolisoquinoline (**1a**) (19.03 μL, 0.1 mmol), phenylacetylene (**2a**) (10.20 μL, 0.1 mmol), JNM-8-AA (1.82 mg, 0.0025 mmol) in CH_3CN (1mL) was stirred at room temperature under photo-irradiation for 12h using 12W white LED. Then the supernatant was analyzed by gas chromatography-mass spectrometry (GC–MS). The reaction conversion was calculated based on the alkyne substrate. After evaporation of the solvent, the crude residue was purified by flash column chromatography on silica gel (petroleum ether : ethyl acetate = 10: 1) to afford desired pure CDC products. Different catalysts, solvents, catalyst loading, and others were investigated in a similar procedure

6.2 Characterization for the product of JNM-8-AA catalyzed CDC reaction.

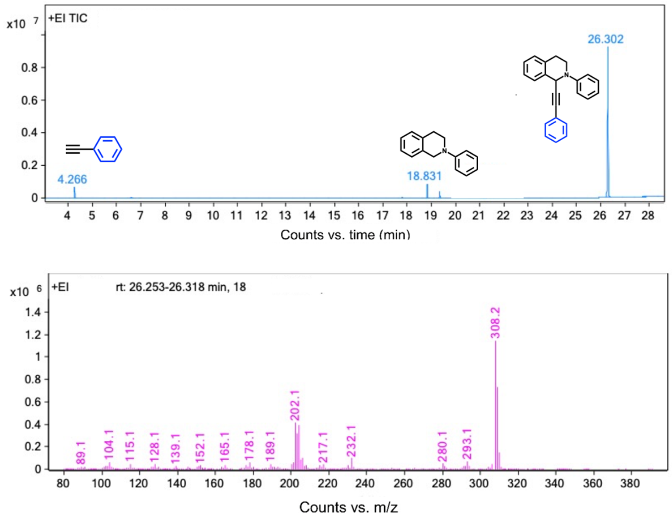

Supplementary Fig 38. Gas chromatography-mass spectrometry (GC–MS) for the reaction solution of JNM-8-AA catalyzed CDC reaction (Top, GC chromatogram; Bottom, EI-MS of product **3a**).

6.3 Gram-scale CDC reactions.

2-Phenyl-1,2,3,4-tetrahydrolisoquinoline (**1a**) (1g, 5 mmol), Phenylacetylene (**2a**) (510 mL, 5 mmol), JNM-8-AA (5 mg, 6.9×10^{-3} mmol) in CH_3CN (AR) (50 mL) were added into a vial. The mixture was stirred under 12W white LED for 12h at room temperature. The conversion of **3a** was monitored by GC-MS. After the reaction was completed, the CDC product **3a** was isolated following the general procedure.

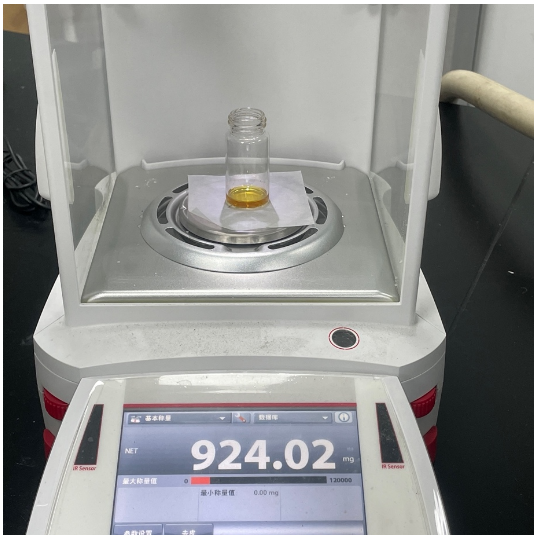

Supplementary Fig 39. The CDC product of **3a** using low loading of JNM-8-AA.

6.4 Comparison of yield and TOF for JNM-8-AA with existing catalysts.

Supplementary Table 13. Comparison of yield and TOF for JNM-8-AA (based on one Cu-CTU (three Cu ions) with previous reports for 2-phenyl-1,2,3,4-tetrahydroisoquinoline and phenylacetylene CDC reactions.

Catalyst	Condition	Additive	Yield	TOF/h^{-1}	Reference
CuOTf	0 °C, O_2, visible light, 48h	Rose Bengal, ligand	81%	0.15	6
CuI	air, visible light, 36h	Rose Bengal	73%	0.20	7
CuO–Fe_3O_4	Air, 50 °C, 3d	None	63%	0.24	8
CuOTf	Air, 50 °C, 2d	Ligand, ^{t}BuOOH	63%	0.28	9
CuCl	Ar, 50 °C, 24h	^{t}BuOOH	86%	0.35	10
$CuCl_2 \cdot 2H_2O$	O_2, rt,17h	^{t}BuOOH	89%	0.52	11
$CuBr_2$	O_2, rt, 3 W blue LED, 12 h	Conjugated polymetric photocatalysts (CPPs)	66%	1.10	12
CuNPs/ZY	Air, 70°C,20 h	^{t}BuOOH -H_2O	62%	2.07	13
$(MeCN)_4P_6$	Air, rt, 5W visible light, 36h	$[Ru(bpy)_2(dtbbp)](PF_6)_2$	88%	5.5	14
DA-CMP3	O_2 (1.0 bar), 30 W blue LED, 25 °C, 1h	None	70%	10.78	15
JNM-8-AA	**Air, 12W white LED, 12h**	**None**	**60%**	**36.23**	**This work**

6.5 Catalytic recyclability for JNM-8-AA.

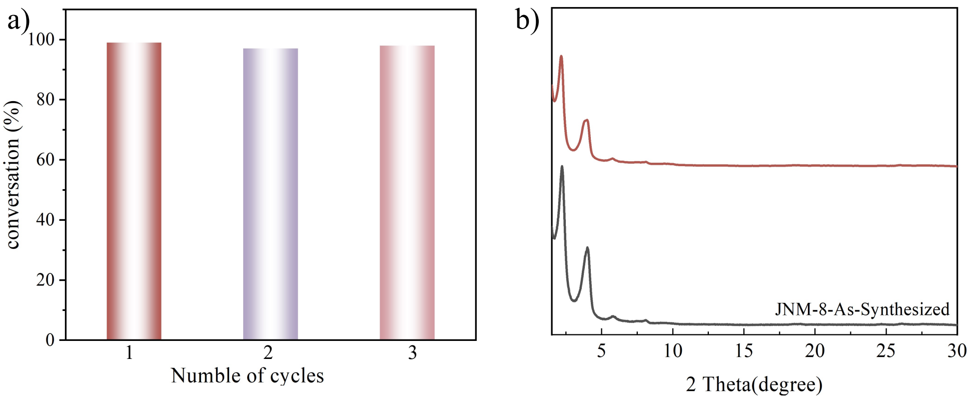

Supplementary Fig 40. (a) Recyclability of JNM-8-AA for CDC reaction. (b) Comparison of PXRD patterns of the As-synthesized JNM-8-AA catalysts and that after being used for 3 cycles.

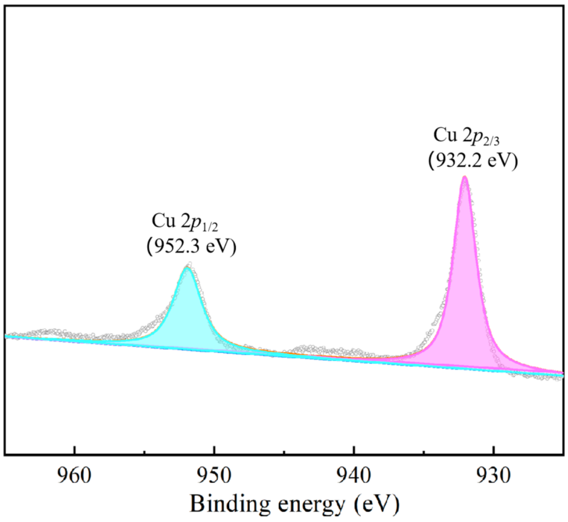

Supplementary Fig 41. XPS for JNM-8-AA after the catalytic reaction.

6.6 Reaction mechanisms.

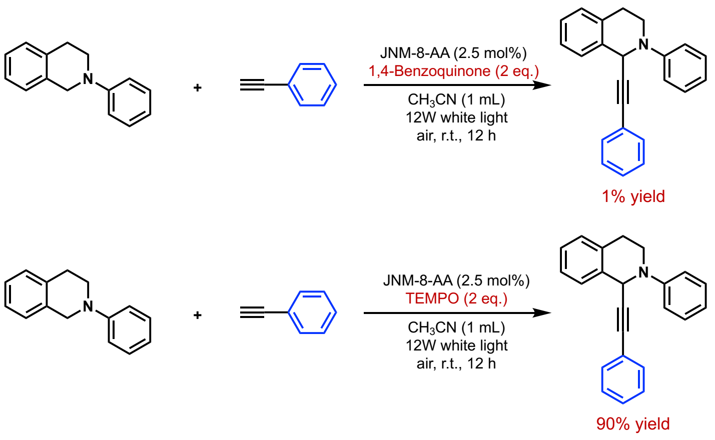

Supplementary Fig 42. Photocatalytic reaction mechanisms verification.

Based on experimental results and related literature a plausible mechanism is proposed.[7, 15] Under visible light irradiation, JNM-8-AA produces the excited species JNM-8-AA*, which is reductively quenched by 2-Phenyl-1,2,3,4-tetrahydrolisoquinoline (**1a**) through a single electron transfer (SET) process to form a radical cation **A** and a radical anion JNM-8-AA$^{\bullet-}$. The generated radical anion JNM-8-AA$^{\bullet-}$ is subsequently quenched by molecular O_2 to form the superoxide anion $O_2^{\bullet-}$and simultaneously regenerate JNM-8-AA. The superoxide anion $O_2^{\bullet-}$ subsequently extracts a proton from **A**, yielding iminium intermediate **B** and releasing HO_2^-. The imine intermediate **B** undergoes addition with copper alkynyl to obtain the final product **3a**.

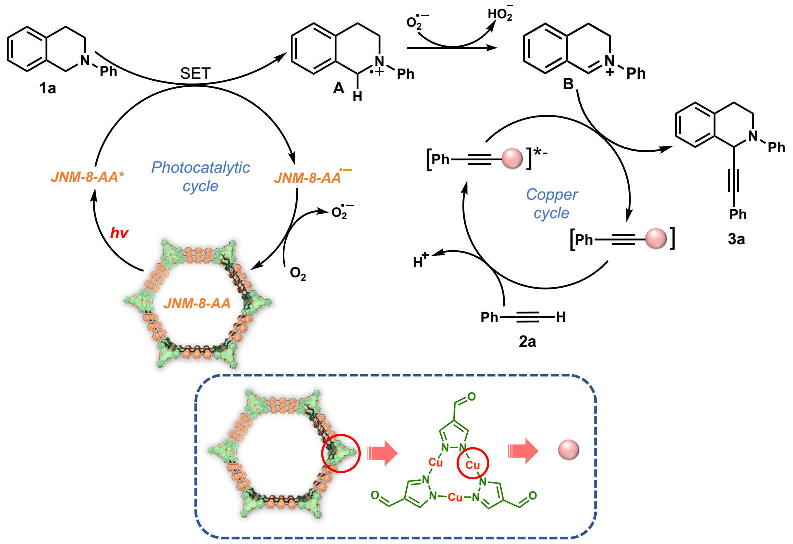

Supplementary Fig 43. Proposed mechanism for the photocatalytic reaction of JNM-8-AA.

6.7 Scope of JNM-8-AA Catalyst for CDC reactions.

2-Phenyl-1-phenylethynyl-1,2,3,4- tetrahydroisoquinoline (3a). ^{1}H NMR (400 MHz, CDCl$_3$) δ[ppm] = 7.43 – 7.27 (m, 6H), 7.24 (s, 5H), 7.15 (d, J = 8.2 Hz, 2H), 6.91 (t, J = 7. 2 Hz, 1H), 5.66 (s, 1H), 3.77 (dd, J = 17.0, 4.7 Hz, 1H), 3.73 –3.63 (m, 1H), 3.18 (dd, J = 14.2, 8.1 Hz, 1H), 3.05 – 2.95 (m, 1H). ^{13}C NMR (101 MHz, CDCl$_3$, 298 K) δ [ppm] = 149.40, 135.29, 134.33, 131.75, 129.16, 128.94, 128.09, 128.06, 127.44, 127.27, 126.31, 122.96, 116.84, 88.43, 84.92, 77.25, 52.45, 43.57, 28.87.

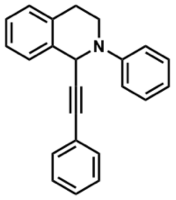

1-(p-Tolylethynyl)-2-phenyl-1,2,3,4-tetrahydroisoquinoline (3b). ^{1}H NMR (400 MHz, CDCl$_3$) δ[ppm] = 7.49 – 7.37 (m, 3H), 7.36 – 7.24 (m, 5H), 7.21 (d, J = 7.1 Hz, 2H), 7.10 (d, J = 6.6 Hz, 2H), 6.97 (t, J = 6.9 Hz, 1H), 5.73 (s, 1H), 3.79 (dtt, J = 19.7, 9.8, 5.0 Hz, 2H), 3.22 (dt, J = 14.9, 7.3 Hz, 1H), 3.05 (d, J = 16.0 Hz, 1H), 2.36 (s, 3H).^{13}C NMR (101 MHz, CDCl$_3$, 298 K) δ [ppm] = 149.60, 138.13, 135.58, 134.43, 131.69, 129.19, 128.90, 127.51, 127.24, 126.32, 119.66, 116.76, 87.89, 84.96, 52.38, 43.52, 28.98.

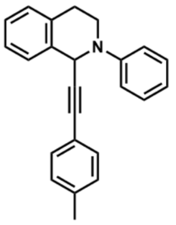

1-(4-Fluorophenylethynyl)-2-phenyl-1,2,3,4-tetrahydroisoquinoline (3c). ^{1}H NMR (400 MHz, CDCl$_3$) δ[ppm] = 7.43 – 7.29 (m, 5H), 7.28 – 7.23 (m, 3H), 7.21 (t, J = 6.4 Hz, 2H), 7.03 – 6.89 (m, 3H), 5.68 (s, 1H), 3.84 – 3.76 (m, 1H), 3.76 – 3.66 (m, 1H), 3.22 (ddd, J = 16.0, 10.0, 6.0 Hz, 1H), 3.04 (dt, J = 16.2, 3.7 Hz, 1H).^{13}C NMR (101 MHz, CDCl$_3$, 298 K) δ [ppm] = 163.61, 134.19, 133.67, 133.59, 129.20, 128.96, 127.40, 126.39, 117.06, 115.47, 115.25, 52.66, 43.81, 28.72.

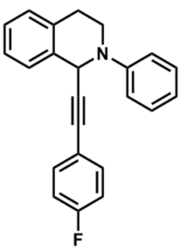

2-Phenyl-1-(-3-Chloro-phenylethynyl)-1,2,3,4-tetrahydroisoquinoline (3d). ^{1}H NMR (400 MHz, CDCl$_3$) δ[ppm] = 7.43 – 7.29 (m, 4H), 7.28 – 7.14 (m, 5H), 7.10 (d, J = 7.7 Hz, 1H), 6.99 (dt, J = 12.2, 7.9 Hz, 3H), 5.68 (s, 1H), 3.80 (dt, J = 9.3, 5.4 Hz, 1H), 3.70 (td, J = 12.4, 11.3, 4.2 Hz, 1H), 3.21 (ddd, J = 16.2, 10.1, 5.9 Hz, 1H), 3.03 (dt, J = 16.2, 3.7 Hz, 1H).^{13}C NMR (101 MHz, CDCl$_3$, 298 K) δ [ppm] = 134.22, 129.70,

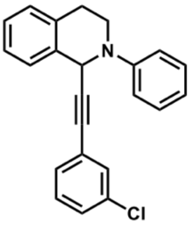

129.62, 129.22, 128.98, 127.64, 127.45, 127.39, 126.41, 118.66, 118.43, 117.08, 115.58, 115.37, 43.78, 29.73, 28.73.

2-phenyl-1-(1-pentynyl)-1,2,3,4-tetrahydroisoquinoline (3e). ^{1}H NMR (400 MHz, $CDCl_3$) δ[ppm] = 7.35 – 7.31 (m, 2H), 7.27 – 7.16 (m, 4H), 7.10 (d, J = 7.9 Hz, 1H), 7.02 (d, J = 7.9 Hz, 1H), 6.92 – 6.84 (m, 1H), 4.45 (s, 1H), 3.78 – 3.70 (m, 1H), 3.66 – 3.58 (m, 2H), 3.14 (td, J = 10.3, 5.2 Hz, 1H), 3.04 – 2.94 (m, 2H), 2.11 (td, J = 7.0, 2.1 Hz, 1H), 1.61 (s, 1H), 1.53 – 1.31 (m, 2H), 0.88 (t, J = 7.4 Hz, 2H).^{13}C NMR (101 MHz, $CDCl_3$, 298 K) δ [ppm] = 129.21, 129.04, 128.83, 128.53, 127.31, 126.96, 126.55, 126.34, 126.13, 126.03, 119.31, 118.68, 116.55, 115.16, 51.75, 50.76, 46.55, 43.19, 29.14, 28.90, 22.17, 20.77, 13.38.

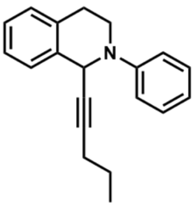

2-Phenyl-1-(1-Nonynyl)-1,2,3,4-tetrahydroisoquinoline (3f). ^{1}H NMR (400 MHz, $CDCl_3$) δ[ppm] = 7.34 (td, J = 7.5, 3.4 Hz, 3H), 7.23 (ddt, J = 13.0, 9.1, 3.9 Hz, 3H), 7.12 (d, J = 8.1 Hz, 2H), 6.91 (t, J = 7.3 Hz, 1H), 5.47 (s, 1H), 3.75 (ddd, J = 11.8, 5.4, 3.6 Hz, 1H), 3.63 (ddd, J = 12.3, 10.5, 4.2 Hz, 1H), 3.14 (ddd, J = 16.2, 10.3, 6.0 Hz, 1H), 2.98 (dt, J = 16.1, 3.7 Hz, 1HS), 2.14 (td, J = 7.0, 1.9 Hz, 2H), 1.42 (dd, J = 13.7, 6.5 Hz, 2H), 1.35 – 1.18 (m, 9H), 0.92 (t, J = 7.0 Hz, 3H).^{13}C NMR (101 MHz, $CDCl_3$, 298 K) δ [ppm] =149.62, 136.23, 134.15, 129.05, 128.84, 127.33, 126.98, 126.15, 116.59, 85.38, 43.22, 31.76, 28.92, 28.67, 22.63, 18.78, 14.15.x

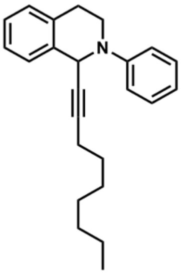

2-Phenyl-(1-cyclopropyl-ethynyl)-1,2,3,4-tetrahydroisoquinoline (3g). ^{1}H NMR (400 MHz, $CDCl_3$) δ[ppm] = 7.35 – 7.29 (m, 3H), 7.24 – 7.17 (m, 3H), 7.07 (d, J = 7.9 Hz, 2H), 6.89 (t, J = 7.3 Hz, 1H), 5.40 (s, 1H), 3.72 (ddd, J = 12.2, 5.9, 3.6 Hz, 1H), 3.60 (ddd, J = 12.3, 10.4, 4.3 Hz, 1H), 3.11 (ddd, J = 16.2, 10.3, 5.9 Hz, 1H), 2.98 – 2.91 (m, 1H), 1.17 (dddd, J = 13.2, 8.2, 5.0, 1.6 Hz, 1H), 0.72 – 0.64 (m, 2H), 0.58 – 0.50 (m, 2H). ^{13}C NMR (101 MHz, $CDCl_3$, 298 K) δ [ppm] = 134.20, 129.03, 128.83, 127.31, 126.97, 126.14, 119.31, 116.50, 88.29, 76.72, 51.73, 43.21, 8.34, 1.05.

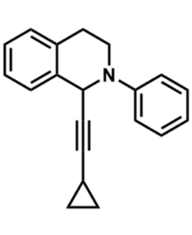

2-Phenyl-(1-trimethylsilanylethynyl)-1,2,3,4-tetrahydroisoquinoline (3h). ^{1}H NMR (400 MHz, $CDCl_3$) δ[ppm] = 7.35 (t, J = 7.0 Hz, 3H), 7.31 – 7.16 (m, 4H), 7.12 (d, J = 7.9 Hz, 2H), 6.93 (t, J = 7.2 Hz, 1H), 5.47 (s, 1H), 3.73 (d, J = 12.1 Hz, 1H), 3.64 (dt, J = 11.9, 6.7 Hz, 1H), 3.15 (ddd, J = 16.0, 10.1, 6.0 Hz, 1H), 2.98 (d, J = 16.0 Hz, 1H), 0.12 – 0.10 (m, 8H). ^{13}C NMR (101 MHz, $CDCl_3$, 298 K) δ [ppm] =149.58, 135.23, 134.35, 129.03, 128.91, 127.46, 127.16, 126.22, 119.70, 104.74, 89.08, 52.73, 43.36, 28.82.

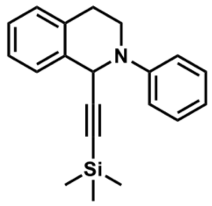

^{1}H NMR and ^{13}C NMR spectra of all compounds

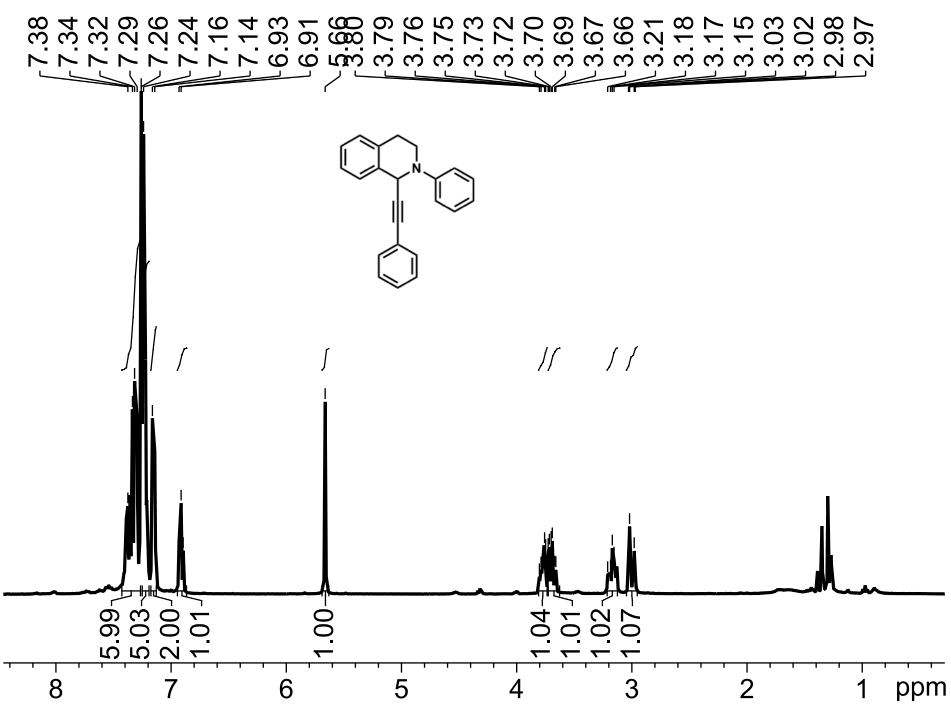

^{1}H NMR spectrum (400 MHz, $CDCl_3$, 298 K) of Compound **3a**

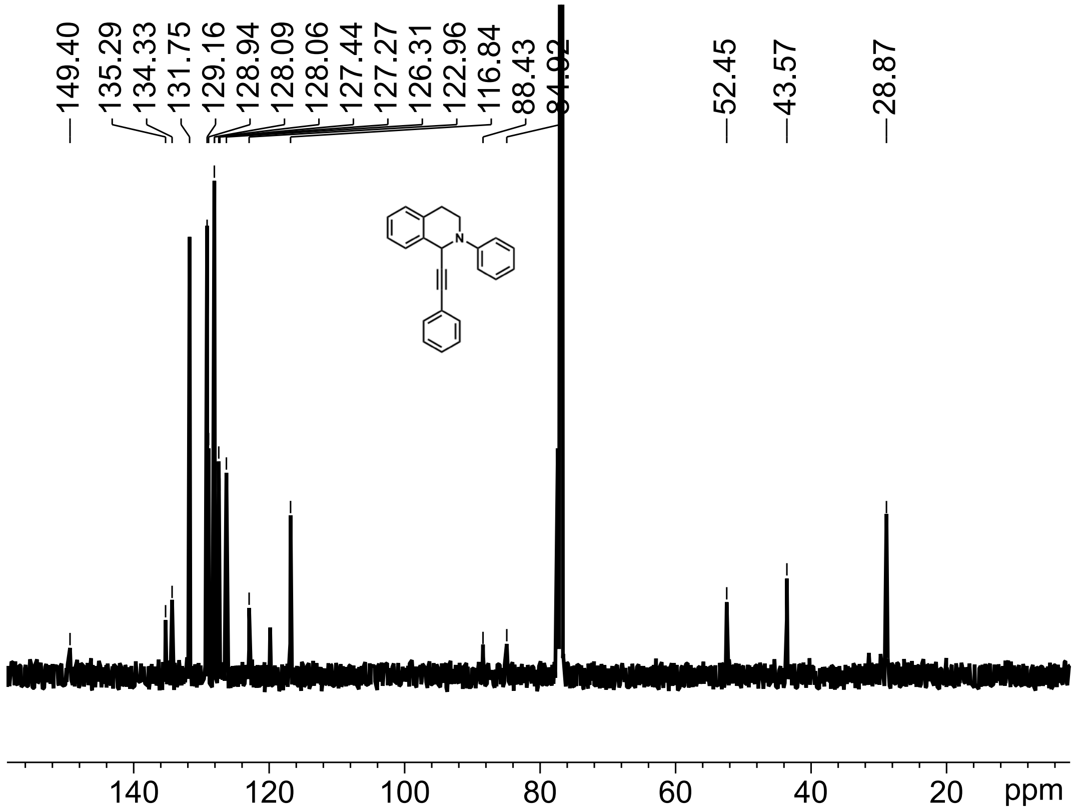

^{13}C NMR spectrum (101 MHz, $CDCl_3$, 298 K) of Compound **3a**

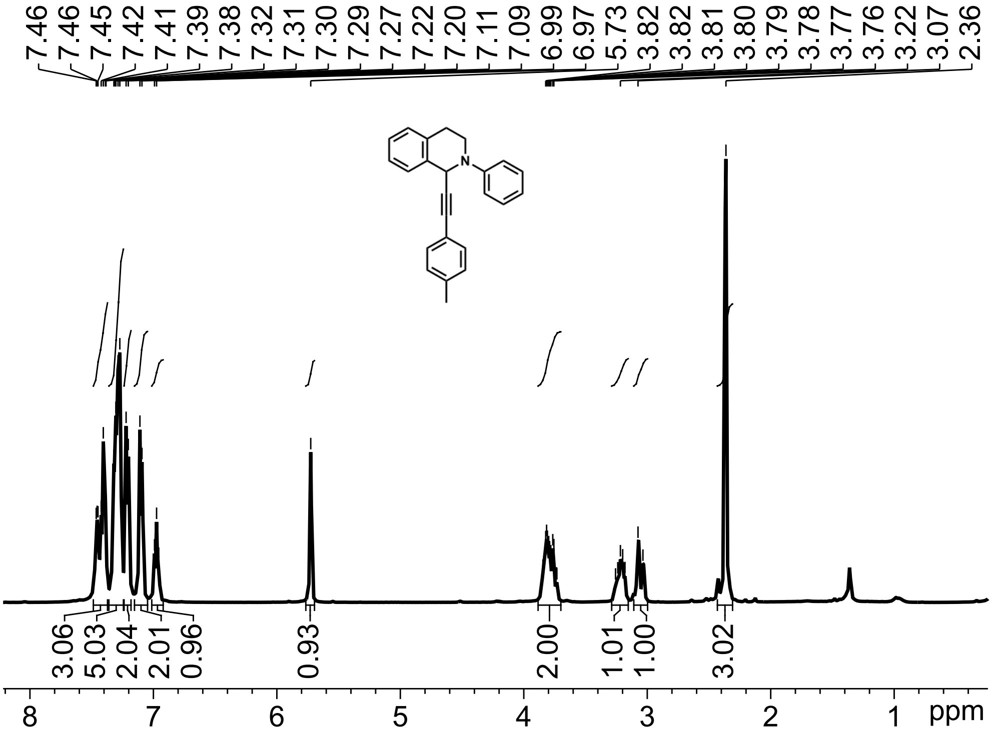

^{1}H NMR spectrum (400 MHz, $CDCl_3$, 298 K) of Compound **3b**

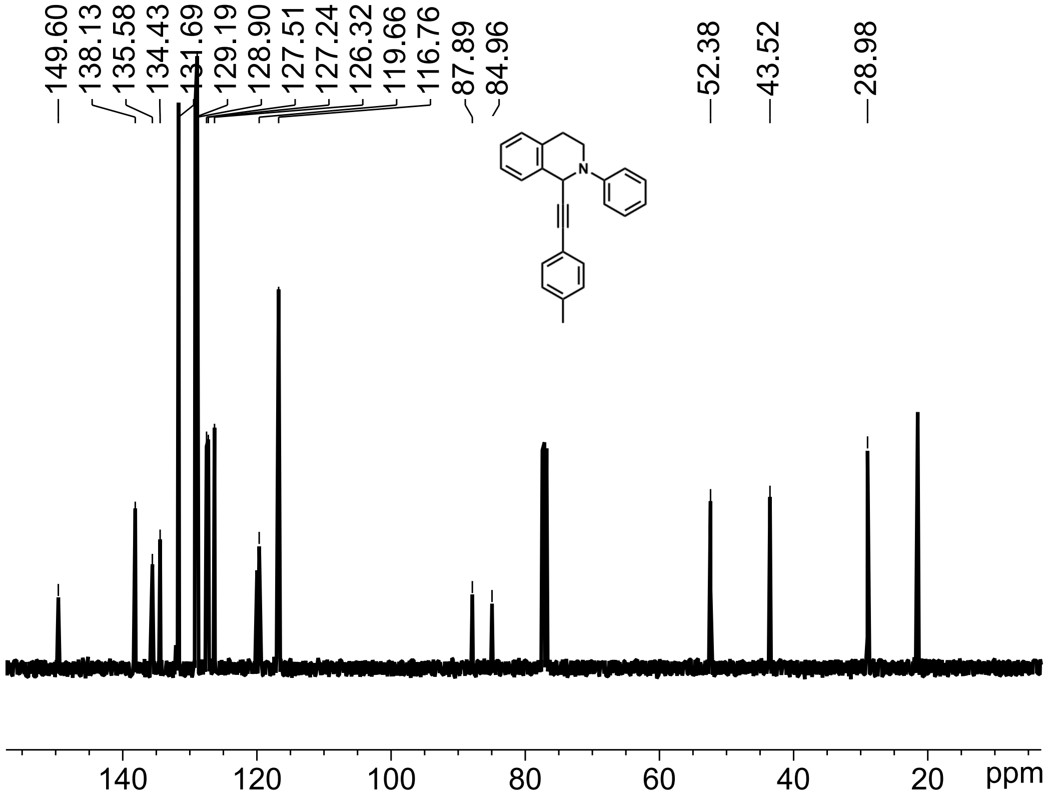

^{13}C NMR spectrum (101 MHz, $CDCl_3$, 298 K) of Compound **3b**

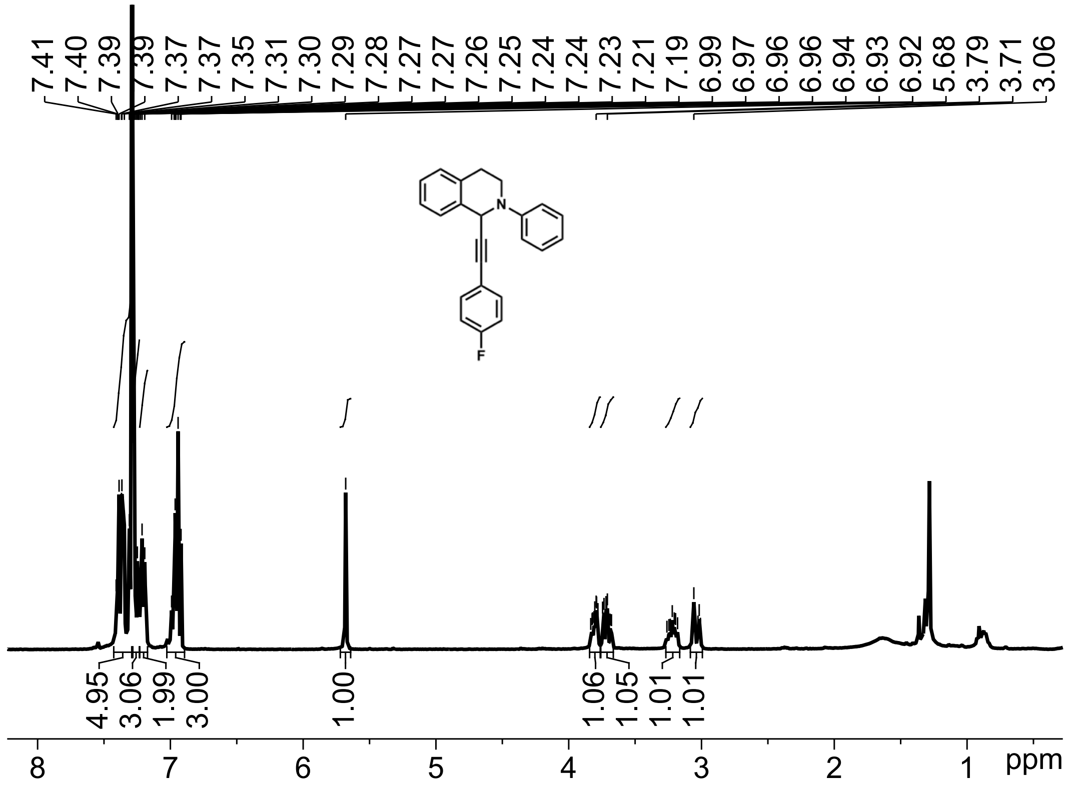

^{1}H NMR spectrum (400 MHz, $CDCl_3$, 298 K) of Compound **3c**

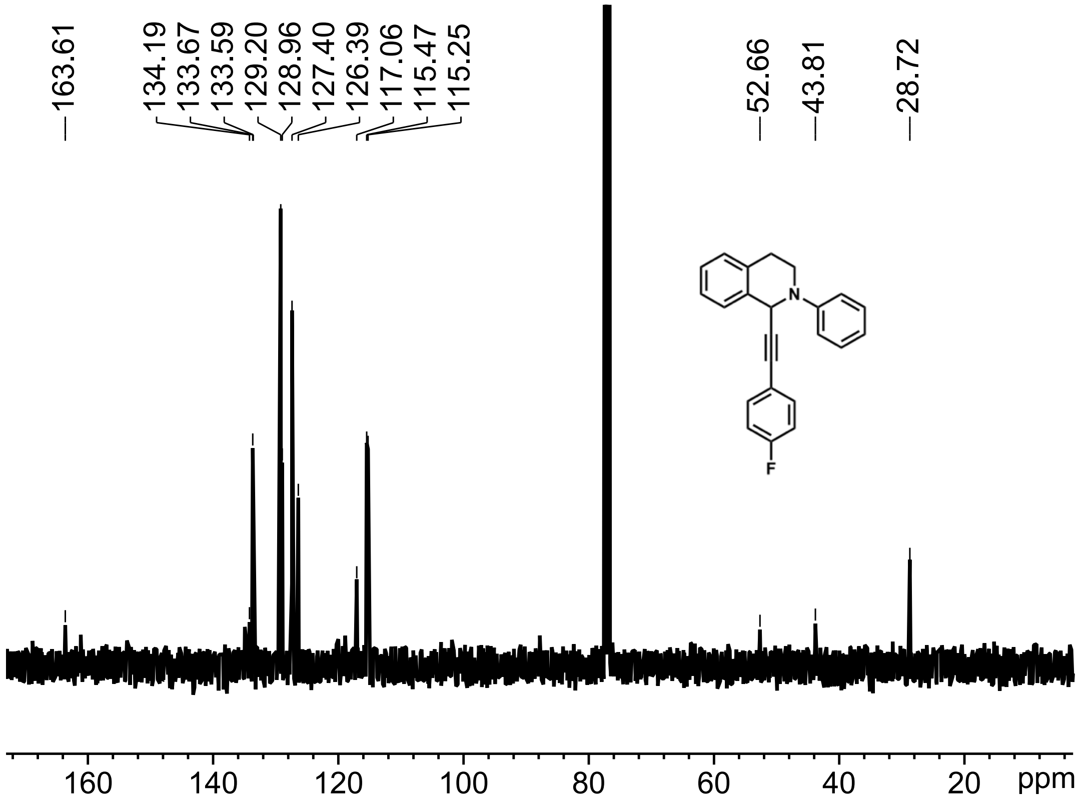

^{13}C NMR spectrum (101 MHz, $CDCl_3$, 298 K) of Compound **3c**

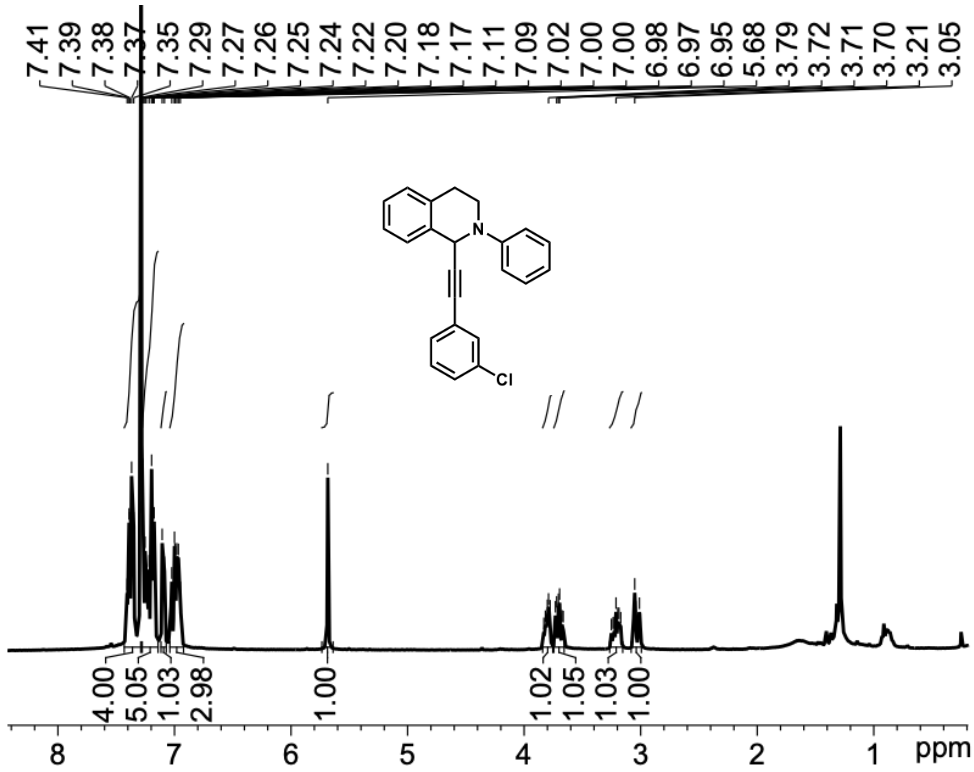

^{1}H NMR spectrum (400 MHz, $CDCl_3$, 298 K) of Compound **3d**

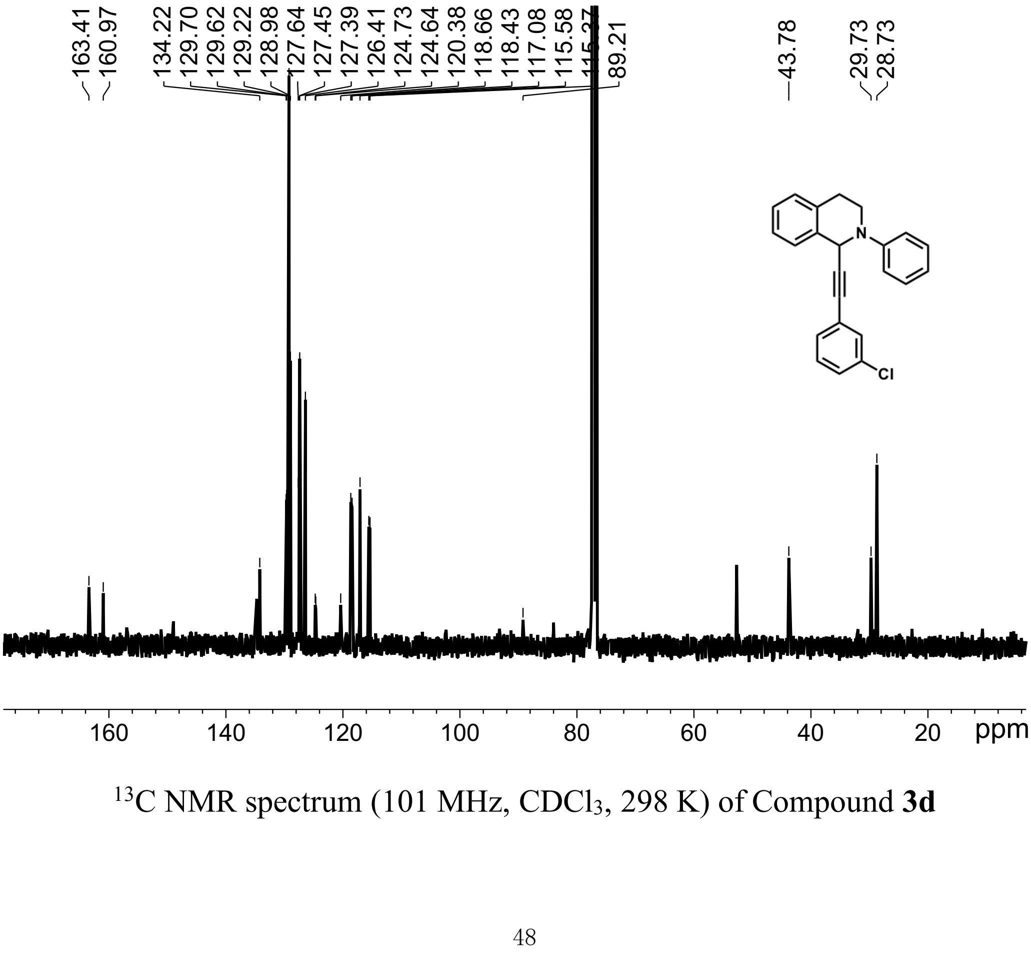

^{13}C NMR spectrum (101 MHz, $CDCl_3$, 298 K) of Compound **3d**

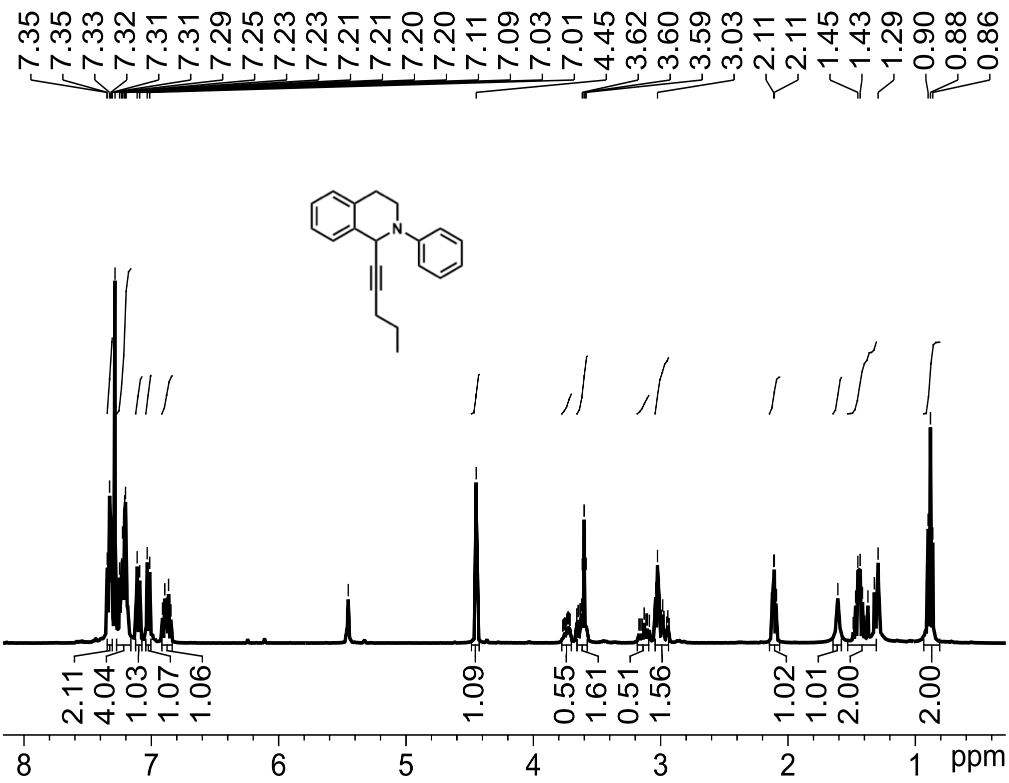

^{1}H NMR spectrum (400 MHz, $CDCl_3$, 298 K) of Compound **3e**

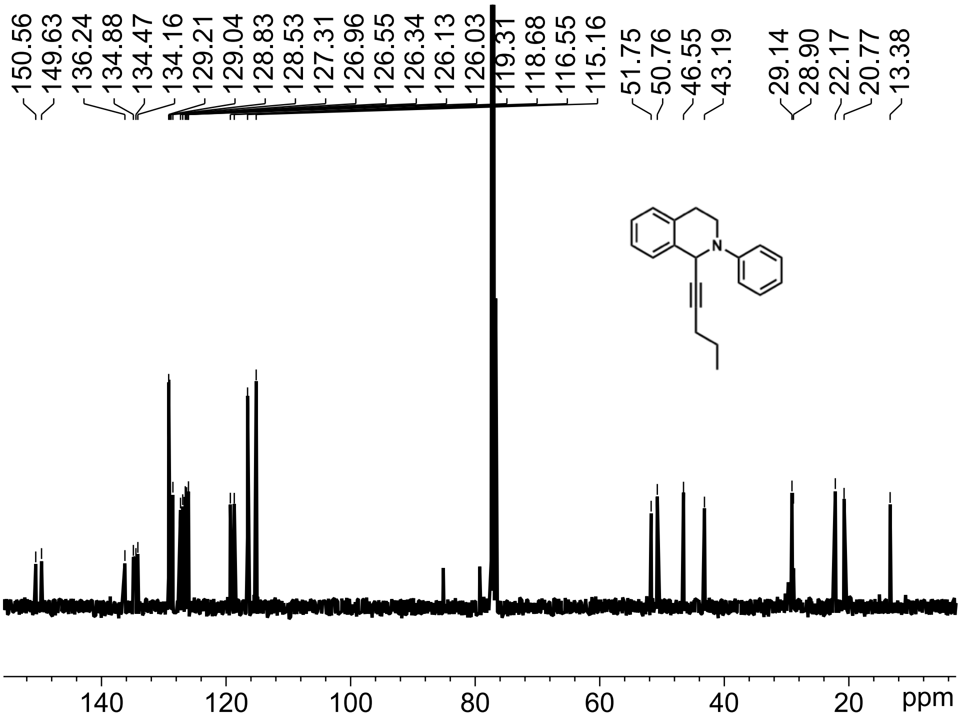

^{13}C NMR spectrum (101 MHz, $CDCl_3$, 298 K) of Compound **3e**

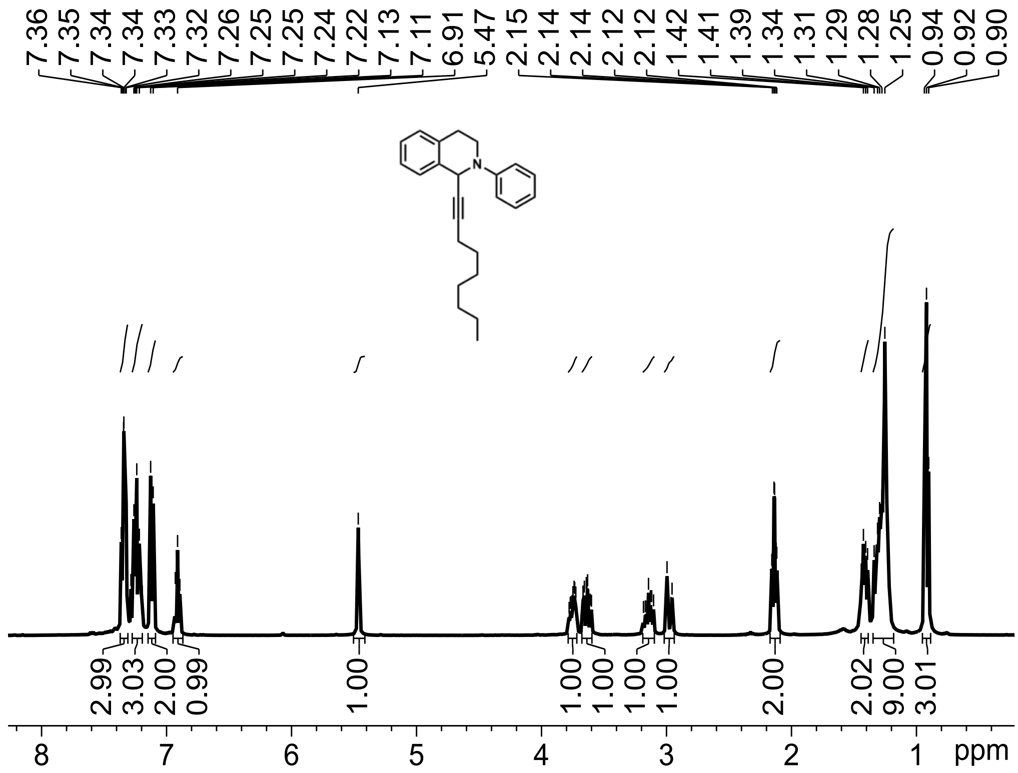

^{1}H NMR spectrum (400 MHz, $CDCl_3$, 298 K) of Compound **3f**

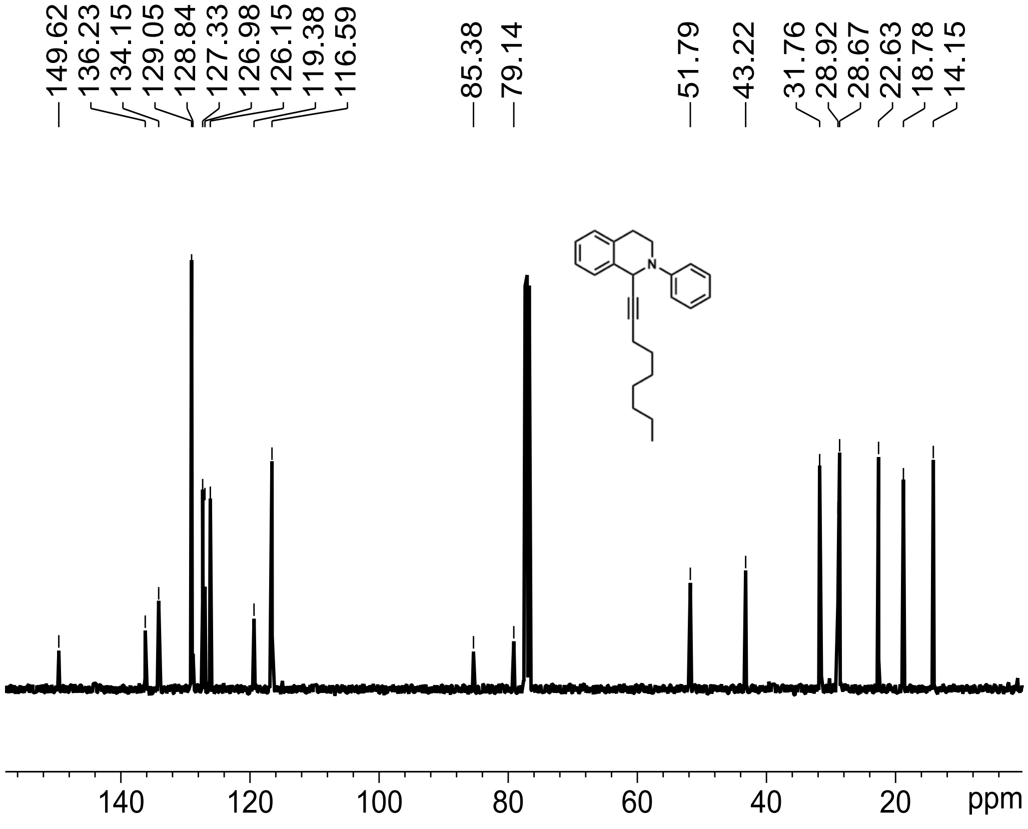

^{13}C NMR spectrum (101 MHz, $CDCl_3$, 298 K) of Compound **3f**

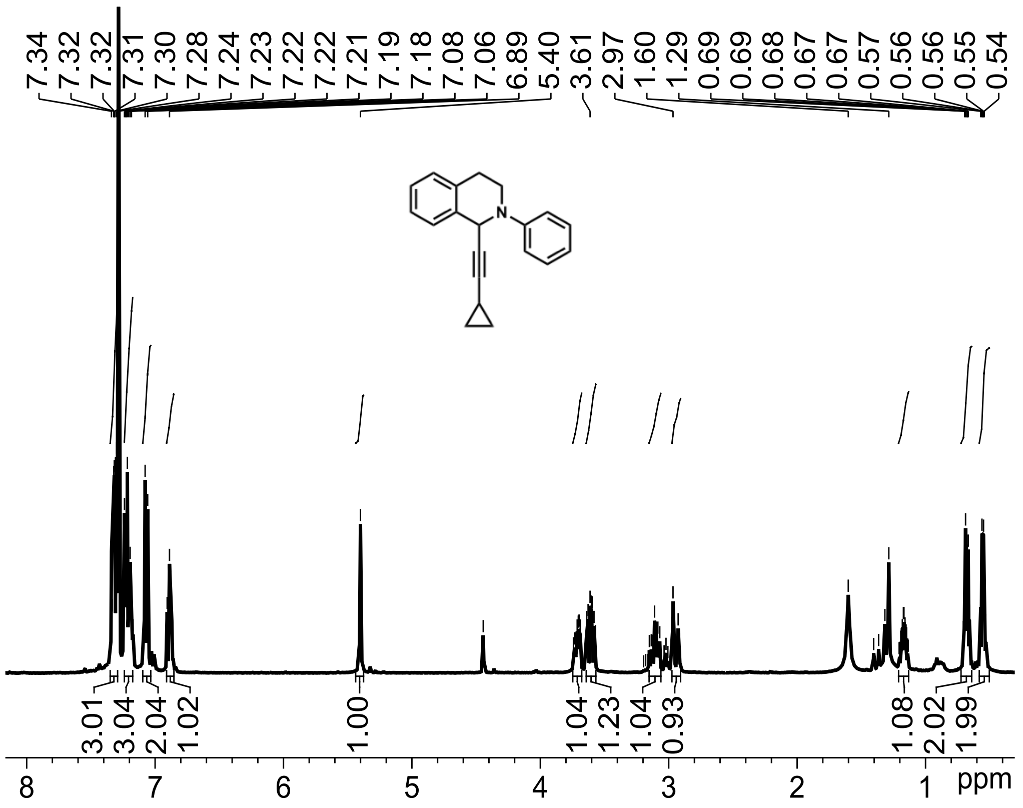

^{1}H NMR spectrum (400 MHz, $CDCl_3$, 298 K) of Compound **3g**

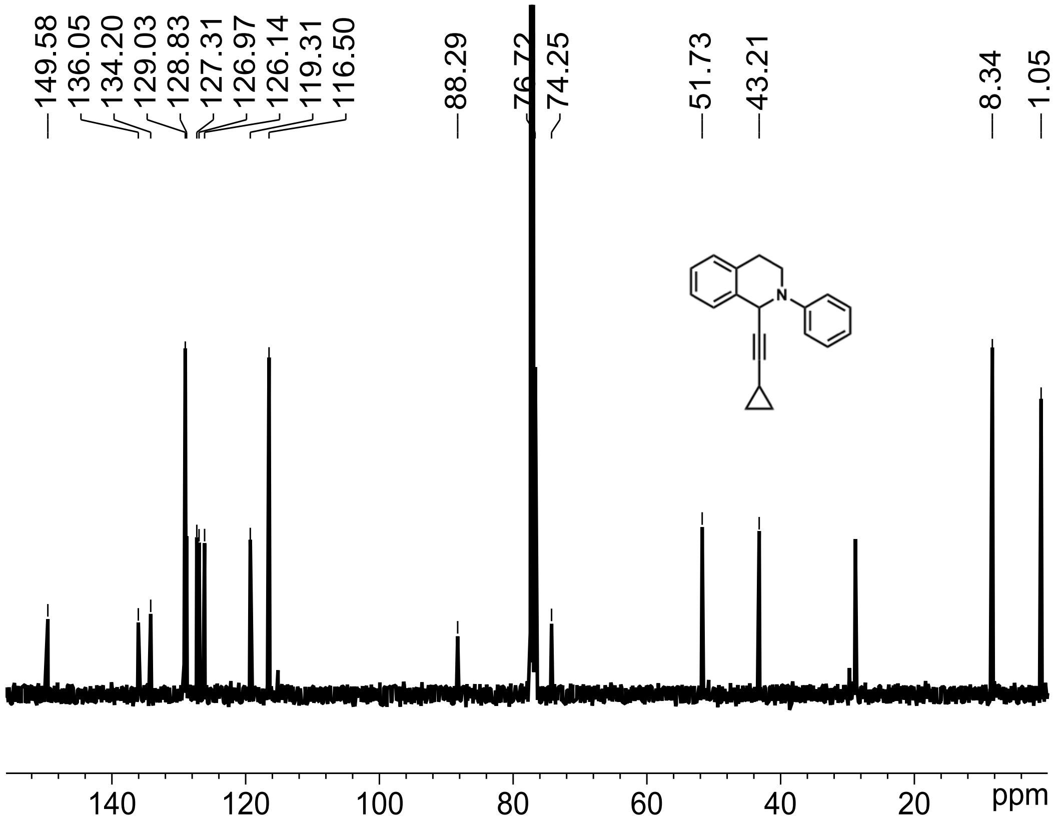

^{13}C NMR spectrum (101 MHz, $CDCl_3$, 298 K) of Compound **3g**

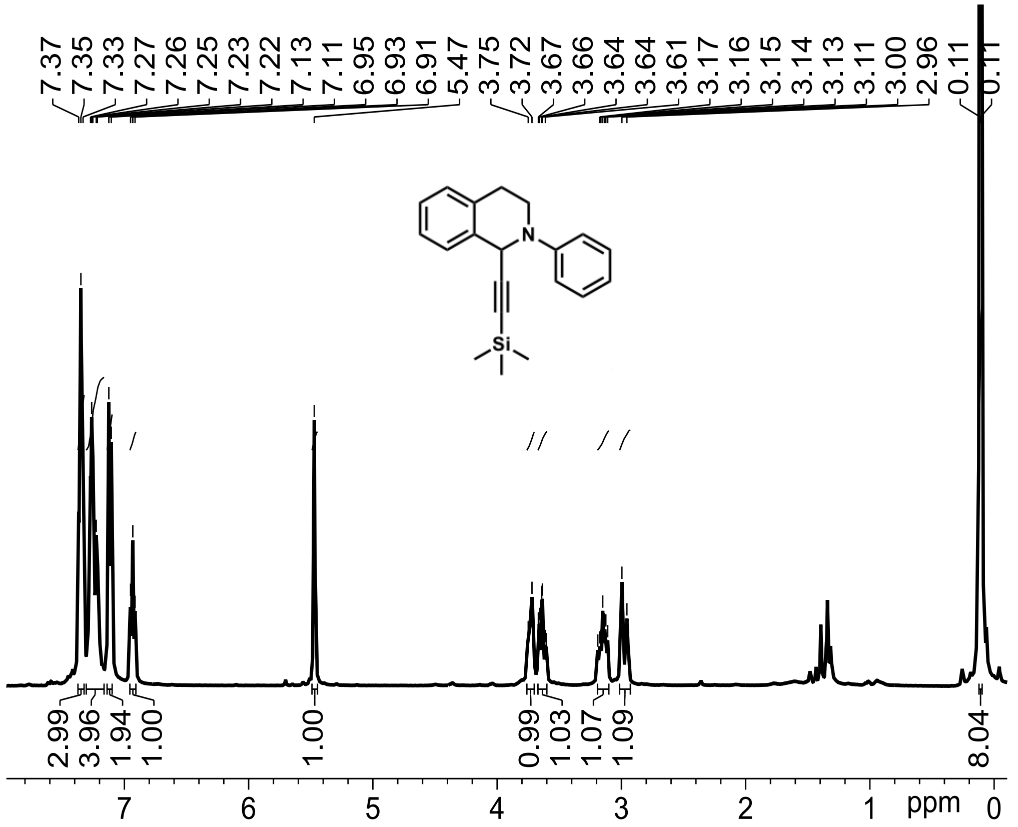

^{1}H NMR spectrum (400 MHz, $CDCl_3$, 298 K) of Compound **3h**

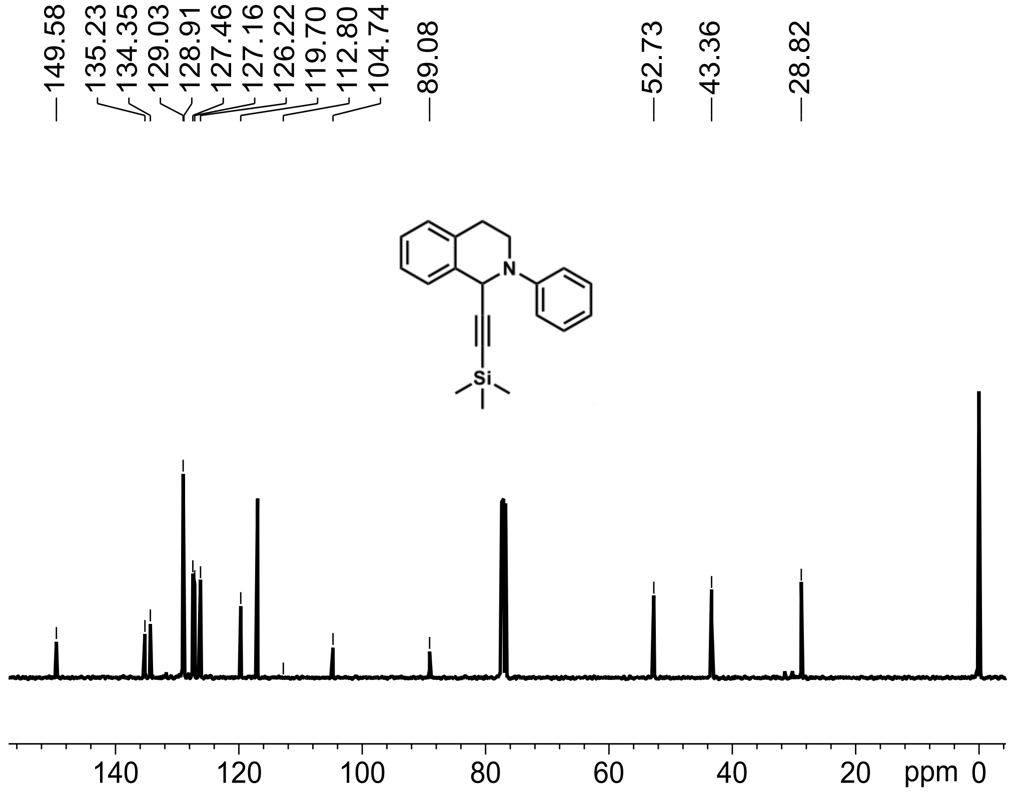

^{13}C NMR spectrum (101 MHz, $CDCl_3$, 298 K) of Compound **3h**

Supplementary references

1. Wei R-J, Zhou H-G, Zhang Z-Y, Ning G-H, Li D. Copper (I)–Organic Frameworks for Catalysis: Networking Metal Clusters with Dynamic Covalent Chemistry. *CCS Chem* **3**, 2045-2053 (2021).
2. Kang C*, et al.* Interlayer Shifting in Two-Dimensional Covalent Organic Frameworks. *J Am Chem Soc* **142**, 12995-13002 (2020).
3. Wu X, Han X, Liu Y, Liu Y, Cui Y. Control Interlayer Stacking and Chemical Stability of Two-Dimensional Covalent Organic Frameworks via Steric Tuning. *J Am Chem Soc* **140**, 16124-16133 (2018).
4. Huang T, Long M, Huo B. Competitive Binding to Cuprous Ions of Protein and BCA in the Bicinchoninic Acid Protein Assay. *Open Biomed Eng J* **4**, 271-278 (2010).
5. Au - Desjardins P, Au - Hansen JB, Au - Allen M. Microvolume Protein Concentration Determination using the NanoDrop 2000c Spectrophotometer. *JoVE*, e1610 (2009).
6. Kumar G, Verma S, Ansari A, Noor-ul HK, Kureshy RI. Enantioselective cross dehydrogenative coupling reaction catalyzed by Rose Bengal incorporated-Cu (I)-dimeric chiral complexes. *Catal Commun* **99**, 94-99 (2017).
7. Fu W, Guo W, Zou G, Xu C. Selective trifluoromethylation and alkynylation of tetrahydroisoquinolines using visible light irradiation by Rose Bengal. *J Flurorine Chem* **140**, 88-94 (2012).
8. Marset X, Pérez JM, Ramón DJ. Cross-dehydrogenative coupling reaction using copper oxide impregnated on magnetite in deep eutectic solvents. *Green Chem* **18**, 826-833 (2016).
9. Li Z, Li C-J. Catalytic enantioselective alkynylation of prochiral sp3 C− H bonds adjacent to a nitrogen atom. *Org Lett* **6**, 4997-4999 (2004).
10. Gröll B, Schaaf P, Schnürch M. Improved simplicity and practicability in copper-catalyzed alkynylation of tetrahydroisoquinoline. *Monatsh Chem* **148**, 91-104 (2017).
11. Boess E, Schmitz C, Klussmann M. A comparative mechanistic study of Cu-catalyzed oxidative coupling reactions with N-phenyltetrahydroisoquinoline. *J Am Chem Soc* **134**, 5317-5325 (2012).
12. Zhu S-S, Liu Y, Chen X-L, Qu L-B, Yu B. Polymerization-Enhanced Photocatalysis for the Functionalization of C (sp3)–H Bonds. *ACS Cata* **12**, 126-134 (2021).
13. Alonso F, Arroyo A, Martín-García I, Moglie Y. Cross-Dehydrogenative Coupling of Tertiary Amines and Terminal Alkynes Catalyzed by Copper Nanoparticles on Zeolite. *Adv Synth Catal* **357**, 3549-3561 (2015).
14. Rueping M, Koenigs RM, Poscharny K, Fabry DC, Leonori D, Vila C. Dual Catalysis: Combination of Photocatalytic Aerobic Oxidation and Metal Catalyzed Alkynylation Reactions—C-C Bond Formation Using Visible Light. *Chem Eur J* **18**, 5170-5174 (2012).

15. Zhi Y*, et al.* Construction of donor-acceptor type conjugated microporous polymers: A fascinating strategy for the development of efficient heterogeneous photocatalysts in organic synthesis. *Appl Catal B Environ* **244**, 36-44 (2019).
